# Zoonotic pathogens in equids in Central Europe: a systematic review

**DOI:** 10.1186/s12917-025-04915-5

**Published:** 2025-07-08

**Authors:** Aisha Arshad, Anna Helga Reif, Jessika-Maximiliane V. Cavalleri, Amélie Desvars-Larrive

**Affiliations:** 1 Centre for Food Science and Veterinary Public Health, Clinical Department for Farm Animals and Food System Science, University of Veterinary Medicine Vienna, Vienna, Austria; 2 Clinical Centre for Equine Health and Research, Clinical Department for Small Animals and Horses, University of Veterinary Medicine Vienna, Vienna, Austria; 3 Complexity Science Hub, Vienna, Austria

**Keywords:** Zoonotic disease, Public health, Equids, Central Europe, Collaboration network

## Abstract

**Background:**

Equids serve diverse roles in contemporary society. Their use as companion animals, in sports, transportation, and food production brings them into close contact with humans, creating potential zoonotic risks. This review had two objectives: (i) to catalogue zoonotic pathogens detected in equids across Central Europe, and (ii) to analyse research trends and collaborations in equid zoonosis studies within the region. We conducted a systematic literature search following PRISMA guidelines to identify publications on naturally occurring zoonotic pathogens in equids from nine countries: Austria, the Czech Republic, Germany, Hungary, Italy, Liechtenstein, Slovakia, Slovenia, and Switzerland. We searched three databases–PubMed, Scopus, and CABI–yielding 1,435 publications, of which 256 were included in the review, spanning 58 years of research (1964–2022).

**Results:**

Our findings revealed increased publications on equine-associated zoonotic pathogens in the region since the 1990s, reflecting growing global concerns about zoonoses, with a recent surge in research on emerging zoonotic agents. A total of 191 zoonotic pathogens were investigated during the study period, with the top 10 most studied pathogens representing 60.2% of the included literature. Researchers from 24 countries, organised into nine research communities, collaborated on equine zoonotic diseases in the region. Germany, Italy, the Czech Republic, and the USA played pivotal roles in the research network. Additionally, we reported 183 zoonotic taxa potentially infecting equids and humans in Central Europe, of which 79.2% were bacteria, 15.8% were eukaryotes, and 4.9% were viruses. This expanded list marks a notable increase from the 56 pathogens reported in previous studies. Pairwise comparisons revealed that Italy and Germany shared the highest number of pathogens (40 taxa), followed by Italy and Switzerland (25 taxa), and Germany and Switzerland (25 taxa).

**Conclusions:**

This study offers an updated list of zoonotic pathogens in equids in Central Europe, highlights emerging threats such as West Nile virus, and underscores the importance of continued surveillance and cross-border collaboration to mitigate these risks.

**Supplementary Information:**

The online version contains supplementary material available at 10.1186/s12917-025-04915-5.

## Background

Humans have domesticated horses for millennia, forming a complex and close relationship across various societies. Historically, horses were central to transportation, agriculture, and warfare, particularly until the end of World War I, and were valued for their meat, hair, and leather. Despite a decline in their population and daily use in higher-income countries, where they are primarily kept for leisure and require stable-based care [1], horses remain indispensable in low- and middle-income countries. In these regions, many people still depend on horses for food, transportation, and income [2]. In 2022, the global equid population was estimated at ~120 million animals, comprising 60.6 million horses (*Equus caballus*) [3], 51.7 million donkeys (*Equus asinus*) [3], and 7.7 million mules (*Equus asinus* x *Equus caballus*) [3, 4]. Europe is home to approximately seven million equids, most of which are horses [5]. France and Germany have the largest equid populations, each exceeding one million horses [6, 7], while Austria is home to approximately 120,000 horses, with 75% of them kept in agricultural holdings [8].

Zoonotic diseases, i.e., diseases naturally transmitted between animals and humans [9], pose serious health risks and significantly impact economies and livelihoods [10–12]. For example, in 1994, an outbreak of Hendra virus–a Henipavirus within the family Paramyxoviridae–in Queensland, Australia, resulted in the deaths of 14 horses and one man [13, 14]. In 2022, West Nile virus (WNV) infections were among the most severe zoonotic diseases affecting humans in Europe, with a case fatality rate of 8.3%, second only to listeriosis [15]. Notably, the 2022 human infection rate in the EU was 0.26 per 100,000 population (corresponding to 1,133 cases). In equids, nine Member States reported 166 WNV cases, representing a 3.5-fold increase from 2021; Italy accounted for the majority of locally acquired infections in both humans and equids across Europe [15].

Zoonotic threats related to equids include both endemic and emerging infections, as well as additional challenges at human-equid interfaces, such as antimicrobial resistance and food safety. These diseases are commonly transmitted at interfaces where humans, equids (and their derived products), arthropod vectors, and the environment interact [16]. Humans can acquire zoonotic infections through various routes [17]: (i) direct contact with an infected animal or biological material (e.g., blood, saliva, urine, or faeces); (ii) indirect transmission, where humans come into contact with infectious agents via contaminated objects, surfaces, the environment, or aerosols in the air [18]; (iii) vector-borne transmission, where arthropods like ticks, mosquitoes, or fleas transmit pathogens through their bites; and (iv) ingestion of contaminated food or water. Individuals who frequently interact with equids–such as equestrians and equine professionals, including veterinarians, farriers, horse trainers, jockeys, breeders, farmers, carriage drivers, and slaughterhouse workers–face a heightened risk of zoonotic infections. They may be exposed to zoonotic agents through direct contact, environmental interactions, or contaminated equipment [19–21].

Building on previous research that identified 56 zoonotic pathogens transmitted from horses worldwide [22], this study aimed to address two primary objectives: (i) to systematically compile and synthesize data on investigated and detected zoonotic pathogens in equids (horses, donkeys, mules, and asses) across Central Europe, broadening the scope of prior research [22] to encompass all equid species, arthropod vectors, and human cases associated with equids; and (ii) to analyse research trends and collaborative networks in equid zoonotic disease studies within the region, identifying thematic shifts, research prioritization patterns, key contributors, and international collaborations. This dual focus allowed us to update the list of zoonotic threats while contextualizing the scientific efforts addressing them. Although Austria was our primary focus, we expanded our investigation to include its eight neighbouring countries: the Czech Republic, Germany, Hungary, Italy, Liechtenstein, Slovenia, Slovakia, and Switzerland. This approach allows for a more comprehensive analysis of regional research efforts and provides a broader epidemiological perspective on equine zoonotic agents in Central Europe.

## Methods

### Pre-registration of the study

This study was registered in PROSPERO (https://www.crd.york.ac.uk/prospero, registration number: CRD42023380399). We made several amendments to the registered protocol. First, due to the magnitude of bacterial studies reporting findings based on isolates rather than animals, the review question was refined to focus on the presence or absence of equine zoonotic pathogens, rather than prevalence data. Second, with respect to risk factors for horse-to-human transmission, many studies did not specify the source of transmission, leading to the exclusion of this data. Lastly, due to the absence of consistent reporting across studies, specific dates on disease onset or emergence could not be determined.

### Literature search strategy

On 13 January 2023, we performed a systematic literature search using three databases: PubMed, Scopus, and CABI. The search query was adapted to each database and included different terms designating members of the taxonomic family Equidae (“Equidae”, “equid”, “equids”, “equine”, “ass”, “asses”, “donkey”, “equus”, “mules”, “mule”, “zebras”, “zebra”, “horse”, “horses”), terms designating the target countries (“Austria”, “Germany”, “Czech*”, “Slovakia”, “Hungary”, “Slovenia”, “Italy”, “Switzerland”, “Liechtenstein”), and the names of known zoonotic agents of horses, as previously defined by Sack et al. [22] (see Additional file 1 for full search strings). The terms were combined using the Boolean operators “AND” and “OR”. We considered the period from the database's inception until the search date. English, German, and French language filters were applied to all databases. The systematic literature search was conducted and reported according to the Preferred Reporting Items for Systematic Review and Meta-Analysis (PRISMA) guidelines [23] (see Additional file 2 for PRISMA Check List).

### Literature screening

We first consolidated the search results from all databases and removed duplicates using Mendeley Reference Manager. The remaining references were then imported into the Rayyan web application [24], where three reviewers independently screened titles and abstracts in “Blind On” mode (English-language records: AA, ADL, JMC; German: JMC; French: ADL). Titles/abstracts were selected if the studies investigated at least one naturally occurring zoonotic taxa (per Sack et al. [22]) in any equine species. Titles/abstracts were included if they were conducted in Austria or its neighbouring countries: the Czech Republic, Germany, Hungary, Italy, Liechtenstein, Slovenia, Slovakia, and Switzerland. Only peer-reviewed journal articles were considered for inclusion. We excluded reviews, conference abstracts, conference proceedings, posters, memorandums, and books. Studies pertaining to experimental trials or tests, vaccine efficacy, diagnostic methods or tools were excluded. Similarly, titles/abstracts that did not present epidemiological outcomes, e.g., prevalence or incidence data, were excluded under the criteria “wrong outcome” in Rayyan.

Full-text articles were screened independently by AA, AHR, and JMC (no French-language studies met the criteria for full-text review). The aforementioned inclusion/exclusion criteria were applied. Disagreements between reviewers were discussed and resolved by consensus.

### Data extraction

For each paper, we manually extracted citation metadata, epidemiological data on the zoonotic pathogen(s) investigated in equids, and corresponding data from other host species when co-investigated with equids. Two reviewers (AA, AHR) extracted and structured the following information into a Microsoft Excel spreadsheet, with AA handling English-language papers and AHR handling German-language papers: (i) article unique ID; (ii) bibliographic data: authors´ names, affiliations, year of publication, journal name, volume, issue, type of paper, and digital object identifier (DOI); (iii) language of the paper; (iv) study objective as mentioned by the authors; (v) country and location of the study; (vi) study design (e.g., cross-sectional, case study) based on Rivas-Ruiz, et al. [25]; (vii) pathogen information: common and scientific name of the pathogen as mentioned by the authors; (viii) host information: common and scientific name of the host species as mentioned by the authors; (ix) type(s) of sample(s) used in the study; (x) condition of the host from which the sample was taken, categorised as “live” if the host was alive at the time of sampling, “dead” if the host was dead during examination, or “biobank” if the samples were archived and not collected specifically for the study. This information was only collected for equids; (xi) detection method(s) employed, (xii) results of the laboratory investigation(s), categorised into “indirect” detection (i.e., the circulation of the zoonotic agents was evidenced through antibody detection) and “direct” detection (i.e., the zoonotic agents was detected in the host). We used the values 0 for negative results and 1 for positive results. When multiple values were applicable in one field, they were separated by semicolons.

We manually classified samples into three categories:"invasive","non-invasive", and"environmental". Invasive samples were obtained via procedures that access internal tissues or fluids such as blood, tissue biopsies, organs, meat samples, and urine collected through catheterisation. Non-invasive samples were collected using methods that do not require direct access to the body’s interior, such as swabs or milk collection. Environmental samples were those obtained from external sources such as surfaces, soil, or arthropod vectors. Faecal samples were classified as invasive if collected directly from the rectum, and as environmental if collected from the ground post-defecation.

### Definition of zoonotic pathogens

When pathogens not listed as zoonotic in Sack et al. [22] were identified in equids within the selected papers, their ability to infect humans (i.e., their zoonotic potential) was assessed through an additional literature review. Building on previous studies [26, 27], we adopted a more inclusive definition of "zoonotic" than the traditional focus on *documented* transmission between animals and humans. This approach was necessary because many transmission pathways (e.g., environmental) remain understudied, and proof of direct transmission often requires longitudinal or experimental data not available for all infectious agents. Our technical definition of a zoonotic pathogen encompassed any pathogens detected in equids with supporting evidence of human infection, including opportunistic and hospital-acquired pathogens. Furthermore, we included vector-borne zoonoses, such as WNV and Usutu virus (USUV), which, although not transmitted *from* equids (equids serve as dead-end hosts), provide valuable surveillance data when detected in equids, informing public health preparedness. Additionally, we included four pathogens that were exclusively documented in humans within our dataset but have been reported in equids in the existing literature. Pathogens reported in equids and described as part of the healthy human microbiome by the authors were excluded from this definition.

Results from this search were entered into a dedicated field (called “zoonotic”), with the values “yes” (indicating the pathogen is confirmed zoonotic based on the list of pathogens from Sack et al. [22] or additional literature), “no” (indicating no evidence of zoonotic potential found in the literature), or “uncertain” (when only the genus of the pathogen was available–preventing a definitive conclusion–or when the literature presented conflicting information). Supporting references on zoonotic potential are provided in Additional file 1.

To maximize the utility of our dataset, we intentionally retained the original taxonomic designations used by study authors, preserving fine-scale classification where available. This approach serves three key purposes: (i) it avoids premature exclusion by capturing understudied taxa with uncertain or emerging zoonotic relevance (e.g., lesser-known species or subspecies); (ii) it enables analytical flexibility, allowing future researchers to aggregate or stratify pathogens according to specific hypotheses; and (iii) it promotes transparency by revealing inconsistencies in taxonomic resolution across studies (such as genus-only versus species-level reporting), which reflect important gaps in surveillance quality.

### Data validation and cleaning

The data underwent a cleaning and validation process. This step was performed in R v.4.4.1. [28].

#### Data cleaning

First, trailing spaces from all fields in the dataset were removed using the *str_trim()* function from the stringr package [29]. To identify inaccuracy, improperly formatted entries, or missing values, we applied the base function *unique().*

#### Laboratory methods standardization

Laboratory detection methods were reported heterogeneously across studies, often using different abbreviations or synonyms for the same technique. To harmonize this information, all method entries were manually reviewed, and synonymous terms were consolidated under a single standardized name (e.g., sero-neutralisation (SN) and virus neutralisation test (VNT) for virus neutralisation test; rRT-PCR and RT-qPCR for reverse transcription quantitative (or real-time) PCR). A mapping of original terms to their harmonized equivalents is provided in the file *“standardization_lab_methods.csv”* [30].

#### Taxonomic validation

To align historical and current nomenclature, resolve taxonomic changes, and ensure the accuracy of scientific and common names for pathogens and hosts, we validated all taxonomic entries against the NCBI Taxonomy database [3] using the R package taxize [31]. Using the *gnr_resolve()* function, we ensured that the scientific names of the pathogens and hosts were up-to-date and correctly spelled. Additionally, we resolved the scientific names corresponding to each host's common name using the NCBI Taxonomic backbone through the *comm2sci()* function. We then retrieved the full taxonomic classification hierarchy for each unique pathogen and host, i.e., superkingdom, order, family, and genus, by querying the NCBI Taxonomy database using the *classification()* function. If the NCBI API did not automatically retrieve a pathogen or host name or their taxonomic classification, we manually imputed it after consulting the NCBI Taxonomy website (https://www.ncbi.nlm.nih.gov/taxonomy).

To compare the genera identified in our study with those reported by Sack et al. [22], we similarly curated the list of zoonotic pathogens from Sack et al. [22] by resolving scientific names and taxonomy using the NCBI database to ensure consistency and accuracy. This step allowed us to standardize their taxa list for comparison with our dataset. We then identified overlapping genera as well as those unique to each study.

#### Pathogen detection and terminology

For this study, we used the following terminology:

- "Detected" refers to pathogens confirmed through either direct (e.g., PCR, culture) or indirect (e.g., serology) methods.
- "Circulating" refers to pathogens detected through direct methods, suggesting current or recent active presence.
- "Investigated" encompasses all pathogens included in the study scope, regardless of detection outcome (i.e., detected and undetected).

### Zoonotic pathogen risk classification

To provide context for our literature-based ranking of zoonotic pathogens, we classified each agent according to three authoritative risk frameworks: (i) the World Health Organization (WHO) Research and Development (R&D) Blueprint for Epidemics [32], including all pathogens listed; (ii) the World Organisation for Animal Health (WOAH) list of notifiable diseases, including all pathogens listed under “Equidae” as well as those classified under “multiple species” [33]; and (iii) the Centers for Disease Control and Prevention (CDC)’s One Health Zoonotic Disease Prioritization (OHZDP) list [34], using genus-level entries (e.g., *Salmonella*, *Echinococcus*) when species-level information was not specified, and species-level entries when available.

### Data analysis and visualisation

Data analysis and visualisation were performed in R v.4.4.1. [28]. Only pathogens identified as zoonotic by Sack et al. [22] or through literature searches were included in the analysis. Pathogens with uncertain zoonotic status at the time of the review were excluded. We determined the yearly publication numbers for each country and the full study region, then plotted these values over time. To visualise publication trends, we fitted a loess regression curve with smoothing parameters of 0.6 or 0.75. This method helps highlight shifts in patterns over time. We calculated the total number of unique zoonotic pathogens investigated across all studies, and the number of detected zoonotic pathogens both regionally and by country. Additionally, we determined the distribution of zoonotic pathogens within the region and individual countries based on direct and indirect detection evidence. All serovars of *Salmonella enterica* subsp. *enterica* were grouped as a single taxon; similarly, serovars of each *Leptospira* species were counted as a single taxon.

We developed a composite risk score to identify zoonotic pathogens of global concern by summing their inclusion across the three priority frameworks (WHO R&D Blueprint, WOAH´s list of notifiable diseases, and CDC´s OHZDP). This resulted in a 0–3 scale, where a score of 3, for example, indicated the agent was recognized as a priority by all three frameworks. To assess discrepancies between internationally recognized risk and research attention (quantified by publication volume), we benchmarked each pathogen’s publication count against the quartiles of the overall publication count distribution. Pathogens with high risk scores (≥ 2) but low publication counts (i.e., at or below the median) were classified as “under-studied”, highlighting potential priority gaps. Conversely, agents with no priority classification (score = 0) but high publication counts (at or above the 75th percentile) were labelled “over-studied”, possibly reflecting research biases or interests less aligned with public health impact.

We used social network analysis, implemented using the R package igraph [35], to explore co-authorship links among scientists and countries in the region. The collaboration network between individuals (or countries) was represented through a graph: $G=(V,E)$, where *V* is a set of vertices, also called nodes, representing co-authors (or countries), connected by *E* edges, also called links [36]. Edges represent co-authorship or co-production between authors (or countries) of a given publication. This network is undirected because co-authorship relationships are inherently symmetric. The graph was weighted based on the number of publications two individuals (or countries) co-authored during the study period, reflecting the intensity (or frequency) of research collaboration. For each node (i.e., author or country), we computed the node degree centrality, which measures the number of connections a node has within the network. This metric provides insight into the influence of a node in the collaboration network. Higher node degree centrality indicates that the author (or country) is involved in more collaborative efforts, suggesting a more central role in the research network. We used the Leiden algorithm [37] to discover the community pattern within the collaboration network among countries. A community within a network is typically defined as a group of nodes with denser connections among themselves than with the rest of the network [38].

Data visualisations were generated with the R packages ggplot2 [39] and ggraph [40]. The map was produced using QGIS v.3.34.9 [41] and Inkscape v.1.3.2., both within a Windows environment.

## Results

### Research trends on zoonotic pathogens in equids of Central Europe

#### Description of the selected studies

**Fig. 1:**
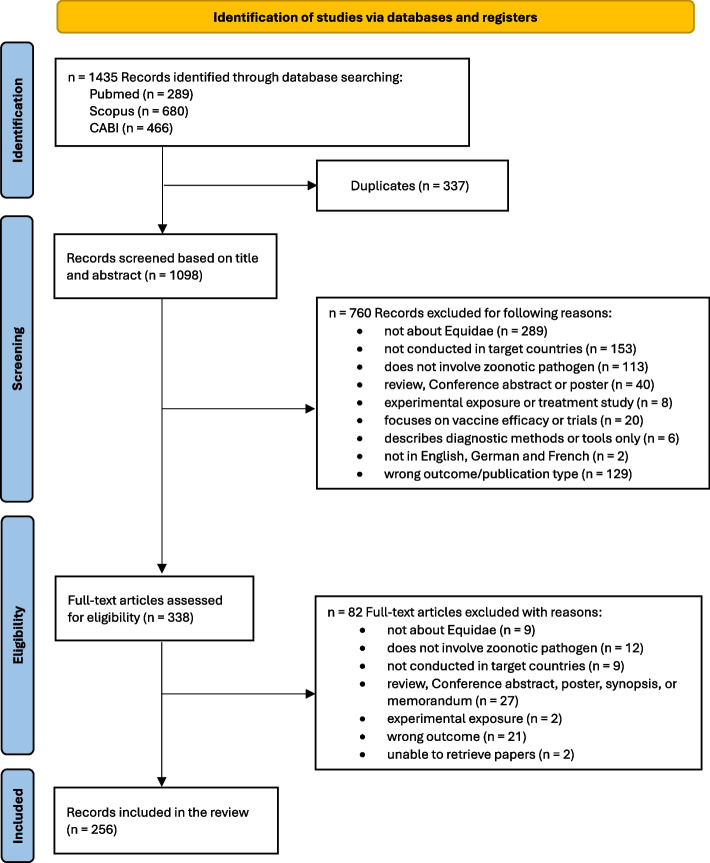
PRISMA flow diagram summarising the search strategy used in this study

The initial search across all databases yielded 1,435 articles (PubMed: 289, Scopus: 680, CABI: 466). After removing duplicates (*n* = 337), 1,098 studies remained for title/abstract screening. Of these, 338 studies were selected for full-text screening. Studies were excluded based on various criteria, with the specifics of these exclusions detailed in Fig. 1. After applying the eligibility criteria, 82 studies were removed, leaving 256 studies that met the inclusion criteria for the systematic literature review, spanning 1964–2022, i.e., 58 years of research on zoonotic pathogens in equids in Central Europe.

Most studies (*n* = 173, 67.6%) were cross-sectional while 48 (18.8%) were case reports, 11 (4.3%) were case series, 13 (5.1%) reported surveillance data, 10 (3.9%) were longitudinal studies, and one was a case–control study (0.4%).

When analysed by country involvement (with multi-country studies counted once for each country they included), Italy was associated with the highest number of studies (*n* = 94, 36.7% of the 256 total), followed by Germany (*n* = 79, 30.9%), Switzerland (*n* = 28, 10.9%), Austria (*n* = 21, 8.2%), the Czech Republic (*n* = 20, 7.8%), Hungary (*n* = 16, 6.2%), Slovakia (*n* = 11, 4.3%), and Slovenia (*n* = 3, 1.2%). No studies involved Liechtenstein. Thirteen studies (5.1%) were multi-country: 11 involved two countries, one included three, and one spanned four countries.

#### Research trends

**Fig. 2:**
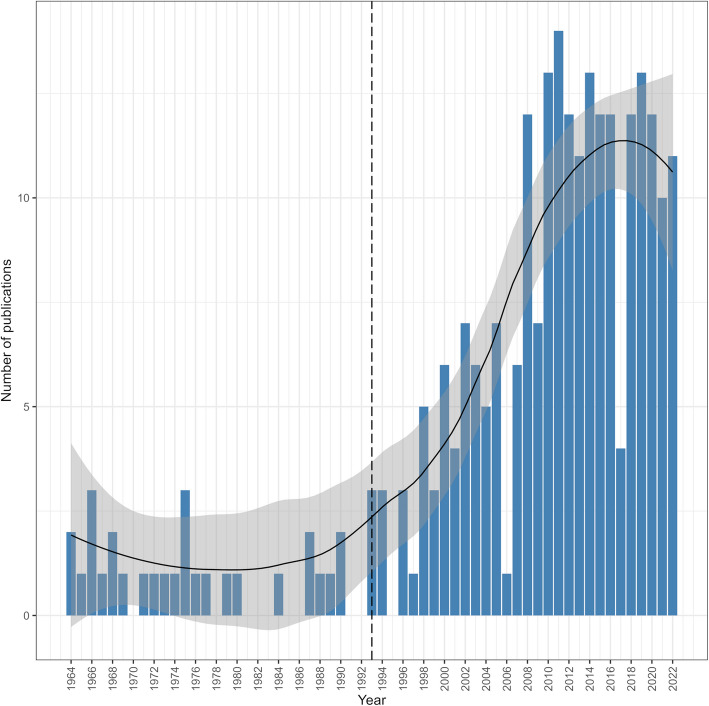
Annual number of publications on zoonotic pathogens in equids across Austria, the Czech Republic, Germany, Hungary, Italy, Slovenia, Slovakia, Liechtenstein, and Switzerland, 1964–2022. The black line represents the loess regression curve using a smoothing parameter of 0.6. The shaded area represents the 95% confidence interval for the fitted loess curve. The dashed line represents the midpoint of the study period. No studies were reported from Liechtenstein

**Fig. 3:**
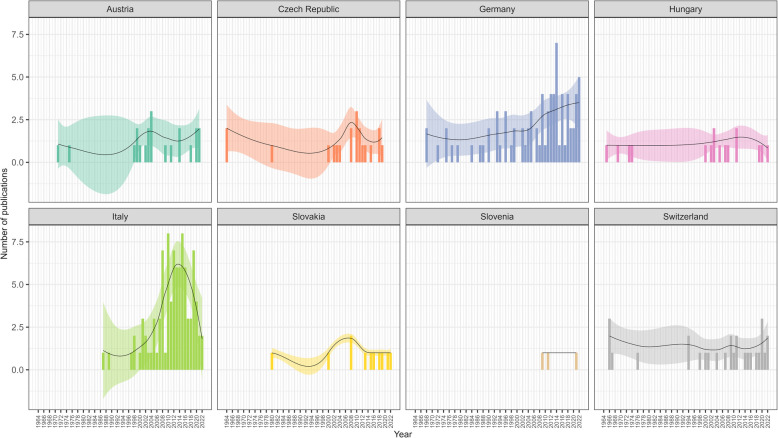
Country-specific annual publication counts on zoonotic pathogens in equids, 1964–2022. The black lines represent the loess regression curves using a smoothing parameter of 0.75. The shaded areas represent the 95% confidence intervals for the fitted loess curves. No studies were reported from Liechtenstein

The annual count of published studies on equine zoonotic diseases remained consistently low until the early 2000s, after which a notable upward trend persisted for approximately 15 years. The number of publications increased from 1964 to 2022, with a 7.1% growth rate between the first and second halves of the study period (Fig. 2). This increase in research interest was also observed at the national level, except for Slovenia, with a notable rise from the late 1990s onwards (Fig. 3). On average, the number of scientific papers on zoonotic pathogens in equids grew by 17.5% per year over the study period, reflecting an exponential trend driven by rapid growth in more recent years.

#### Research-prioritized pathogens across countries

Overall, 191 zoonotic pathogens were investigated in equids between 1964 and 2022 (Table 1), of which 150 (78.5%) were bacteria, 31 (16.2%) were eukaryotes, and 10 (5.2%) were viruses. Of 191 zoonotic agents, 187 were documented in equids in the reviewed literature, and four additional pathogens (*Ehrlichia* spp.*, Ehrlichia chaffeensis*, *Trichophyton verrucosum*, and *Brucella melitensis*) were included because they infect equids [22, 42–44], even though they were only reported in humans in the reviewed studies. Taxonomic resolution varied: 176 pathogens were identified to species/subspecies, while 15 were reported only at the genus level, reflecting inconsistencies in reporting granularity.

**Table 1:** List of 191 zoonotic pathogens investigated in equids across 256 studies conducted in Austria, the Czech Republic, Germany, Hungary, Italy, Slovenia, Slovakia, Liechtenstein, and Switzerland, 1964–2022. The zoonotic agents are ordered according to research interest, indicated by the number of publications. Each agent is also scored based on inclusion in three international risk prioritization frameworks: the World Health Organization (WHO) R&D Blueprint for Epidemics, the World Organisation for Animal Health (WOAH) list of notifiable diseases, and the Centers for Disease Control and Prevention (CDC)’s One Health Zoonotic Disease Prioritization (OHZDP) list. The composite risk score (0–3) reflects the number of frameworks recognizing each agent. No studies were reported from Liechtenstein

NCBI-resolved scientific name of the zoonotic pathogen	Number of publications	Percentage of the total number of publications	WHO R&D Blueprint	WOAH Terrestrial Code	CDC OHZDP	Risk score
West Nile virus	39	15.2	No	Yes	Yes	2
*Anaplasma phagocytophilum*	26	10.2	No	No	No	0
*Escherichia coli*	26	10.2	No	No	No	0
*Leptospira*^1^	20	7.8	No	No	Yes	1
*Staphylococcus aureus*	17	6.6	No	No	No	0
Equine influenza virus H3N8	16	6.2	No	Yes	No	1
*Streptococcus equi* subsp.* zooepidemicus*	16	6.2	No	No	No	0
methicillin-resistant* Staphylococcus aureus*	16	6.2	No	No	No	0
*Borreliella burgdorferi*	15	5.9	No	No	No	0
Usutu virus	14	5.5	No	No	No	0
*Staphylococcus*^1^	13	5.1	No	No	No	0
*Tick-borne encephalitis virus*	13	5.1	No	No	No	0
*Cryptosporidium parvum*	12	4.7	No	No	No	0
*Pseudomonas aeruginosa*	10	3.9	No	No	No	0
Equine influenza virus H7N7	9	3.5	No	Yes	No	1
*Prescottella equi*	9	3.5	No	No	No	0
*Toxoplasma gondii*	9	3.5	No	No	No	0
*Salmonella*^1^	8	3.1	No	No	Yes	1
*Acinetobacter baumannii*	7	2.7	No	No	No	0
*Cryptosporidium*^1^	7	2.7	No	No	No	0
*Klebsiella pneumoniae*	7	2.7	No	No	No	0
*Enterococcus faecalis*	6	2.3	No	No	No	0
*Klebsiella oxytoca*	6	2.3	No	No	No	0
*Salmonella enterica* subsp. *enterica*^2^	6	2.3	No	No	Yes	1
*Staphylococcus xylosus*	6	2.3	No	No	No	0
*Streptococcus dysgalactiae* subsp.* equisimilis*	6	2.3	No	No	No	0
*Streptococcus equi* subsp.* equi*	6	2.3	No	No	No	0
*Clostridioides difficile*	5	2	No	No	No	0
*Cryptosporidium* sp*.* horse genotype	5	2	No	No	No	0
*Halicephalobus gingivalis*	5	2	No	No	No	0
*Klebsiella*^1^	5	2	No	No	No	0
*Mammaliicoccus sciuri*	5	2	No	No	No	0
*Proteus mirabilis*	5	2	No	No	No	0
*Bacillus anthracis*	4	1.6	No	Yes	Yes	2
*Borreliella afzelii*	4	1.6	No	No	No	0
*Citrobacter freundii*	4	1.6	No	No	No	0
*Clostridium perfringens*	4	1.6	No	No	No	0
*Enterococcus*^1^	4	1.6	No	No	No	0
*Fasciola hepatica*	4	1.6	No	No	No	0
*Giardia intestinalis*	4	1.6	No	No	No	0
*Leishmania infantum*	4	1.6	No	Yes	No	1
Lyssavirus rabies	4	1.6	No	Yes	Yes	2
*Mammaliicoccus lentus*	4	1.6	No	No	No	0
*Morganella morganii*	4	1.6	No	No	No	0
*Staphylococcus pseudintermedius*	4	1.6	No	No	No	0
*Trichophyton equinum*	4	1.6	No	No	No	0
*Acinetobacter*^1^	3	1.2	No	No	No	0
*Actinobacillus*^1^	3	1.2	No	No	No	0
*Aeromonas hydrophila*	3	1.2	No	No	No	0
*Borreliella garinii*	3	1.2	No	No	No	0
*Chlamydia psittaci*	3	1.2	No	No	No	0
*Enterococcus casseliflavus*	3	1.2	No	No	No	0
*Enterococcus faecium*	3	1.2	No	No	No	0
*Leptospira interrogans*^2^	3	1.2	No	No	Yes	1
*Neospora caninum*	3	1.2	No	No	No	0
*Pasteurella multocida*	3	1.2	No	No	No	0
*Staphylococcus capitis*	3	1.2	No	No	No	0
*Staphylococcus cohnii* subsp*. cohnii*	3	1.2	No	No	No	0
*Staphylococcus haemolyticus*	3	1.2	No	No	No	0
*Streptococcus equi*	3	1.2	No	No	No	0
*Trichinella spiralis*	3	1.2	No	Yes	No	1
*Trichophyton mentagrophytes*	3	1.2	No	No	No	0
*Acinetobacter calcoaceticus*	2	0.8	No	No	No	0
*Acinetobacter johnsonii*	2	0.8	No	No	No	0
*Actinobacillus equuli*	2	0.8	No	No	No	0
*Alcaligenes faecalis*	2	0.8	No	No	No	0
*Bacillus cereus*	2	0.8	No	No	No	0
*Bartonella henselae*	2	0.8	No	No	No	0
*Burkholderia mallei*	2	0.8	No	Yes	No	1
*Chlamydia abortus*	2	0.8	No	No	No	0
*Clostridium botulinum* B	2	0.8	No	No	No	0
Cowpox virus	2	0.8	No	No	No	0
*Coxiella burnetii*	2	0.8	No	Yes	No	1
*Echinococcus equinus*	2	0.8	No	No	Yes	1
*Echinococcus granulosus*	2	0.8	No	Yes	Yes	2
*Encephalitozoon cuniculi*	2	0.8	No	No	No	0
*Giardia*^1^	2	0.8	No	No	No	0
*Klebsiella aerogenes*	2	0.8	No	No	No	0
*Klebsiella pneumoniae* subsp.* pneumoniae*	2	0.8	No	No	No	0
*Leptospira kirschneri*	2	0.8	No	No	Yes	1
*Mammaliicoccus vitulinus*	2	0.8	No	No	No	0
*Microsporum canis*	2	0.8	No	No	No	0
*Mycobacterium avium* subsp.* paratuberculosis*	2	0.8	No	No	No	0
*Providencia stuartii*	2	0.8	No	No	No	0
*Pseudomonas fluorescens*	2	0.8	No	No	No	0
*Salmonella enterica*	2	0.8	No	No	Yes	1
*Serratia marcescens*	2	0.8	No	No	No	0
Shiga toxin-producing* Escherichia coli*	2	0.8	No	No	No	0
*Staphylococcus auricularis*	2	0.8	No	No	No	0
*Staphylococcus cohnii*	2	0.8	No	No	No	0
*Staphylococcus delphini*	2	0.8	No	No	No	0
*Staphylococcus hominis*	2	0.8	No	No	No	0
*Staphylococcus intermedius*	2	0.8	No	No	No	0
*Staphylococcus succinus*	2	0.8	No	No	No	0
*Stenotrophomonas maltophilia*	2	0.8	No	No	No	0
*Streptococcus dysgalactiae*	2	0.8	No	No	No	0
*Streptococcus equinus*	2	0.8	No	No	No	0
*Trichinella*^1^	2	0.8	No	Yes	No	1
*Trichostrongylus axei*	2	0.8	No	No	No	0
methicillin-resistant* Staphylococcus pseudintermedius*	2	0.8	No	No	No	0
*Acinetobacter lwoffii*	1	0.4	No	No	No	0
*Acinetobacter pittii*	1	0.4	No	No	No	0
*Acinetobacter towneri*	1	0.4	No	No	No	0
*Actinobacillus equuli* subsp.* equuli*	1	0.4	No	No	No	0
*Actinobacillus equuli* subsp.* haemolyticus*	1	0.4	No	No	No	0
*Actinobacillus lignieresii*	1	0.4	No	No	No	0
*Actinobacillus ureae*	1	0.4	No	No	No	0
*Aeromonas bestiarum*	1	0.4	No	No	No	0
*Aeromonas caviae*	1	0.4	No	No	No	0
*Aeromonas eucrenophila*	1	0.4	No	No	No	0
*Aeromonas media*	1	0.4	No	No	No	0
*Bacillus licheniformis*	1	0.4	No	No	No	0
*Bacteroides pyogenes*	1	0.4	No	No	No	0
*Bartonella*^1^	1	0.4	No	No	No	0
*Borreliella lusitaniae*	1	0.4	No	No	No	0
*Borreliella valaisiana*	1	0.4	No	No	No	0
*Brucella abortus*	1	0.4	No	Yes	Yes	2
*Brucella melitensis*^3^	1	0.4	No	Yes	Yes	2
*Burkholderia cenocepacia*	1	0.4	No	No	No	0
*Burkholderia cepacia*	1	0.4	No	No	No	0
*Campylobacter*^1^*	1	0.4	No	No	No	0
*Candida albicans*	1	0.4	No	No	No	0
*Chlamydia pneumoniae*	1	0.4	No	No	No	0
Chlamydia trachomatis*	1	0.4	No	No	No	0
*Citrobacter braakii*	1	0.4	No	No	No	0
Clostridium botulinum*	1	0.4	No	No	No	0
*Clostridium perfringens* A	1	0.4	No	No	No	0
*Clostridium perfringens* C	1	0.4	No	No	No	0
*Cronobacter sakazakii*	1	0.4	No	No	No	0
*Cryptosporidium muris*	1	0.4	No	No	No	0
*Dicrocoelium dendriticum*	1	0.4	No	No	No	0
Echinococcus multilocularis*	1	0.4	No	Yes	Yes	2
*Ehrlichia*^1, 3^	1	0.4	No	No	No	0
*Ehrlichia chaffeensis*^3^	1	0.4	No	No	No	0
*Enterococcus durans*	1	0.4	No	No	No	0
*Enterococcus gallinarum*	1	0.4	No	No	No	0
*Enterococcus gilvus*	1	0.4	No	No	No	0
*Enterococcus malodoratus*	1	0.4	No	No	No	0
*Enterococcus mundtii*	1	0.4	No	No	No	0
*Enterocytozoon bieneusi*	1	0.4	No	No	No	0
*Escherichia fergusonii*	1	0.4	No	No	No	0
*Fusobacterium necrophorum*	1	0.4	No	No	No	0
*Fusobacterium varium*	1	0.4	No	No	No	0
*Geotrichum candidum*	1	0.4	No	No	No	0
*Haemophilus influenzae*	1	0.4	No	No	No	0
*Hafnia alvei*	1	0.4	No	No	No	0
Japanese encephalitis virus*	1	0.4	No	Yes	No	1
*Klebsiella pneumoniae* subsp. ozaenae*	1	0.4	No	No	No	0
*Kosakonia cowanii*	1	0.4	No	No	No	0
*Leishmania*^1^	1	0.4	No	Yes	No	1
*Leptospira borgpetersenii*	1	0.4	No	No	Yes	1
*Listeria monocytogenes*	1	0.4	No	No	No	0
Mammalian orthoreovirus 2	1	0.4	No	No	No	0
Mammalian orthoreovirus 3 Dearing	1	0.4	No	No	No	0
*Microsporum equinum*	1	0.4	No	No	No	0
*Moraxella catarrhalis*	1	0.4	No	No	No	0
*Morganella morganii* subsp.* morganii*	1	0.4	No	No	No	0
*Mycobacterium avium* subsp.* hominissuis*	1	0.4	No	No	No	0
*Nannizzia gypsea*	1	0.4	No	No	No	0
Neorickettsia risticii*	1	0.4	No	No	No	0
*Paraclostridium bifermentans*	1	0.4	No	No	No	0
*Pasteurella caballi*	1	0.4	No	No	No	0
*Pneumocystis carinii*	1	0.4	No	No	No	0
*Pseudescherichia vulneris*	1	0.4	No	No	No	0
*Pseudomonas fulva*	1	0.4	No	No	No	0
*Pseudomonas luteola*	1	0.4	No	No	No	0
*Pseudomonas mendocina*	1	0.4	No	No	No	0
*Pseudomonas oryzihabitans*	1	0.4	No	No	No	0
*Raoultella ornithinolytica*	1	0.4	No	No	No	0
*Raoultella planticola*	1	0.4	No	No	No	0
*Raoultella terrigena*	1	0.4	No	No	No	0
*Rickettsia conorii*	1	0.4	No	No	No	0
*Serratia rubidaea*	1	0.4	No	No	No	0
*Staphylococcus chromogenes*	1	0.4	No	No	No	0
*Staphylococcus epidermidis*	1	0.4	No	No	No	0
*Staphylococcus equorum*	1	0.4	No	No	No	0
*Staphylococcus pasteuri*	1	0.4	No	No	No	0
*Staphylococcus simulans*	1	0.4	No	No	No	0
*Staphylococcus ureilyticus*	1	0.4	No	No	No	0
*Staphylococcus warneri*	1	0.4	No	No	No	0
*Streptococcus agalactiae*	1	0.4	No	No	No	0
*Streptococcus gallolyticus*	1	0.4	No	No	No	0
*Trichophyton verrucosum*^3^	1	0.4	No	No	No	0
*Trichostrongylus*^1^*	1	0.4	No	No	No	0
*Trueperella pyogenes*	1	0.4	No	No	No	0
*Vagococcus fluvialis*	1	0.4	No	No	No	0
*Vibrio vulnificus*	1	0.4	No	No	No	0
*Yersinia enterocolitica*	1	0.4	No	No	No	0
cefotaxime-resistant* Klebsiella michiganensis*	1	0.4	No	No	No	0
cefotaxime-resistant* Klebsiella oxytoca*	1	0.4	No	No	No	0
cefotaxime-resistant* Klebsiella pneumoniae*	1	0.4	No	No	No	0

^1^ Most specific scientific denomination extracted from the information source

^2^ Including all serovars

^3^ Zoonotic agents detected in humans in the region, which can infect equids

^*^ Zoonotic agents that were investigated but not evidenced across the 256 studies

The top 10 most studied zoonotic pathogens in equids in Central Europe were: WNV (39 publications, 15.2% of the total number of included papers), *Anaplasma phagocytophilum*, *Escherichia coli* (each accounting for 26 publications, 10.2%), *Leptospira* (*n* = 20, 7.8%), *Staphylococcus aureus* (*n* = 17, 6.6%), equine influenza virus H3N8, *Streptococcus equi* subsp. *zooepidemicus,* methicillin-resistant *Staphylococcus aureus* (MRSA) (each counting for 16 publications, 6.2%), *Borreliella burgdorferi* (*n* = 15, 5.9%), and USUV (14, 5.5%), which together represent 154 unique publications (60.2% of the included literature).

**Fig. 4:**
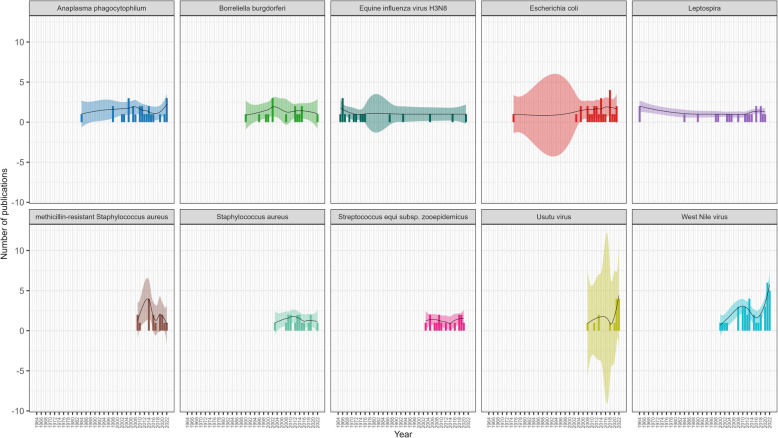
Annual number of publications for the top 10 investigated zoonotic pathogens in equids across Austria, the Czech Republic, Germany, Hungary, Italy, Slovenia, Slovakia, Liechtenstein, and Switzerland, 1964–2022. The black lines represent the loess regression curves using a smoothing parameter of 0.75. The shaded areas represent the 95% confidence intervals for the fitted loess curves. No studies were reported from Liechtenstein

Analysis of the number of annual publications for the top 10 most investigated zoonotic pathogens revealed distinct temporal patterns. *Leptospira* and equine influenza virus H3N8 have been long studied in the region, whereas research on *B. burgdorferi* and *A. phagocytophilum* began midway through the studied period, highlighting a shift in focus to these pathogens. In contrast, WNV, USUV, *S. aureus* (including MRSA), and *S. equi* subsp. *zooepidemicus* have gained research attention more recently (Fig. 4).

#### Mismatches between recognized risk and research effort

**Fig. 5:**
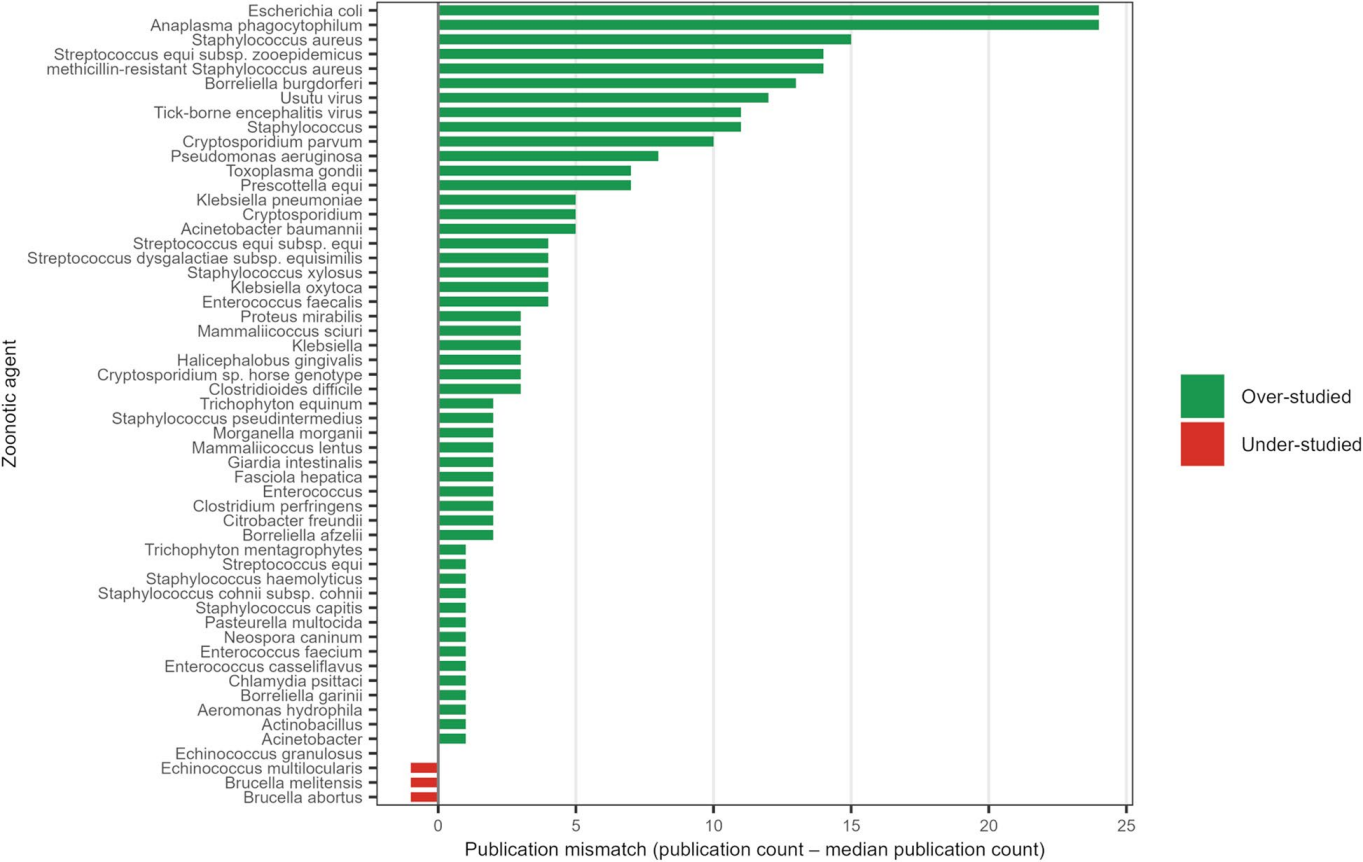
Diverging bar chart of risk-publication mismatch among investigated zoonotic pathogens in equids across Austria, the Czech Republic, Germany, Hungary, Italy, Slovenia, Slovakia, Liechtenstein, and Switzerland, 1964–2022. Agents were scored 0–3 by summing their inclusion across three prioritization frameworks (World Health Organization (WHO) Research and Development (R&D) Blueprint for Epidemics, World Organisation for Animal Health (WOAH) list of notifiable diseases, and the Centers for Disease Control and Prevention (CDC)’s One Health Zoonotic Disease Prioritization (OHZDP)). "Mismatch" identifies pathogens where research attention (publication volume) diverges from risk prioritization score: negative (red) bars indicate under-studied agents (i.e., showing high risk (score ≥ 2) but below- or equal to-median publication counts); positive (green) bars represent over-studied agents that lack formal risk classification (score = 0) but have publication counts in the upper quartile. Pathogens are ordered by magnitude of mismatch

None of the zoonotic agents in equids identified via our literature search was listed in the WHO R&D Blueprint [32], 16 were notifiable to WOAH, and 15 were listed in the CDC OHZDP framework. Among them, seven were included in both the WOAH [33] and CDC [34] priority lists (Table 1). Out of the 191 investigated zoonotic pathogens, we identified four as “under-studied” (high risk score and low research effort estimated based on publication count), whereas 51 were identified as “over-studied” (risk score of 0 and high publication volume) (Fig. 5).

#### Investigated equid hosts across countries

The reviewed studies examined multiple equid hosts for zoonotic agents across the nine central European countries. The most frequently studied species was *Equus caballus* (horse, *n* = 245 publications), followed by *Equus asinus* (ass, 30). Less commonly investigated equids included *Equus quagga chapmani* (Chapman's Zebra, 3), *Equus przewalskii* (Przewalski's horse, 2), and several others such as *Equus asinus somalicus* (Somali Wild Ass), *Equus asinus x Equus caballus* (mule), *Equus grevyi* (Grevy's Zebra), *Equus hemionus kulan* (Kulan), *Equus kiang* (Tibetan Wild Ass*), Equus quagga boehmi* (Grant's Zebra), *Equus quagga borensis* (Selous' Zebra), *Equus quagga burchellii* (Burchell's Zebra), *Equus zebra hartmannae* (Hartmann´s Mountain Zebra), and zebra (scientific name not specified), each with one study.

#### Types of samples and detection methods

The detection methods reported across the 256 included studies spanned a wide range of direct and indirect approaches (Table 2). Molecular assays predominated: PCR was employed in 108 studies (42.2%), with real-time and reverse transcription variants used in an additional 28 studies (10.9%). Sequencing was reported in 21 studies (8.2%), and multilocus sequence typing (MLST) in 17 studies (6.6%). Culture‐based techniques were featured in 80 studies (31.3%), often in combination with identification methods, e.g., MALDI-TOF MS (used in 14 of all 256 studies, 5.5%), pulsed-field gel electrophoresis (PFGE; 10 studies, 3.9%), analytical profile index (API) (nine studies, 3.5%), disk diffusion test (six studies, 2.3%), or Vitek automated system (four studies, 1.6%). Genomic approaches such as targeted sequencing or whole-genome sequencing (WGS) were employed in 21 (8.2%) and six studies (2.3%), respectively.

**Table 2:** Detection methods by zoonotic pathogen taxonomic order across 256 studies on zoonotic diseases in equids conducted in Austria, the Czech Republic, Germany, Hungary, Italy, Slovenia, Slovakia, Liechtenstein, and Switzerland, 1964–2022. No studies were reported from Liechtenstein

Taxonomic order	Standardized method name
Actinomycetales	API, culture
Aeromonadales	API, culture, MALDI-TOF MS
Amarillovirales	CFT, clinical signs, culture, ELISA, HIA, histology, histopathology, IFA, IgG ELISA, IgG PRNT, IgG VNT, IgM ELISA, IHC, NAT, PCR, PRNT, RNA-scope in situ hybridization, RT-PCR, RT-qPCR, sequencing, SN assay, VNT
Apansporoblastina	IHC, PCR
Articulavirales	CFT, culture, HE stain, HIA, histopathology, microscopy, NI, optical immunoassay, pathology, PCR, qPCR, RT-qPCR, serology, SRH, virus isolation
Bacillales	API, biochemical examination, culture, disk diffusion method, identification by conventional method, identification by Vitek automated system, IHC, MALDI-TOF MS, MLST, PCR, PFGE, sequencing, SNRA, spa typing, WGS
Bacteroidales	culture
Burkholderiales	CFT, culture, MALDI-TOF MS, Mallein test, PCR
Campylobacterales	culture
Chitovirales	culture, ELISA, gel electrophoresis, histopathology, microscopy, PCR, PRNT, TEM
Chlamydiales	CFT, IHC, MIFT, PCR, qPCR, sequencing
Cyclophyllidea	electrosyneresis, ELISA, histopathology, necropsy, pathology, PCR, RFLP-PCR, sequencing
Diplomonadida	DFA assay, faecal smear, flotation, IFA, merthiolate-iodine-formaldehyde concentration method, PCR, rapid immunoassay
Dipodascales	culture, microscopy
Enterobacterales	API, biochemical examination, culture, disk diffusion method, ELISA, Giemsa stain, HE stain, histology, identification by rapid ID32E system, identification by Vitek automated system, IHC, Jameson method, lysotype, MALDI-TOF MS, MLST, pathology, PCR, PFGE, qPCR, sequencing, serology, Sven Gard method, WGS
Eubacteriales	culture, identification by conventional method, identification by kit ANAERO, LAT, mouse bioassay, PCR, sequencing
Eucoccidiorida	Baermann technique, culture, DFA assay, DIA, ELISA, faecal smear, flotation, fluorescence microscopy, gel electrophoresis, HE stain, IFA, IgG ELISA, IgG LAT, IHC, larvae migration method, LAT, MAT, microscopy, MLST, PCR, PCR–RFLP, qPCR, rapid immunoassay, RFLP, sedimentation, sequencing, Ziehl-Nielsen stain
Fusobacteriales	culture
Hyphomicrobiales	culture, IFA, PCR
Lactobacillales	API, biochemical examination, culture, disk diffusion method, gel electrophoresis, histopathology, identification by Vitek automated system, IHC, pathology, PCR, RAPD-PCR, sequencing
Legionellales	IgG ELISA, PCR
Leptospirales	agglutinin lysin reaction, culture, ELISA, IFA, IgA ELISA, IgG ELISA, IgM ELISA, IHC, MAT, paper electrophoresis, PCR, qPCR, serology
Lysobacterales	API, culture, identification by Vitek automated system, MALDI-TOF MS, PCR, sequencing
Mononegavirales	fuorescence microscopy, HE stain, histopathology, IFA, IHC, infection trial on mice, microscopy, pathology, PCR, sequencing
Moraxellales	API, culture, identification by Vitek automated system, MALDI-TOF MS, MLST, PCR, PFGE, sequencing, WGS
Mycobacteriales	AGID, API, culture, disk diffusion method, ELISA, flotation, HE stain, histopathology, IHC, larvae migration method, M-VNTR, MLSSR, PCR, PFGE, qPCR, Ziehl–Neelsen stain
Onygenales	API, culture, ELISA, fluoroscent brightener, fuorescence microscopy, Grocott stain, hair perforation test, HE stain, histopathology, lactophenol blue stain, microscopy, nicotinic acid test, PAS, PCR, urease test
Pasteurellales	API, culture, identification by Vitek automated system, MALDI-TOF MS, PCR, sequencing
Peptostreptococcales	culture, ELISA, identification by kit ANAERO, MALDI-TOF MS, MLST, PCR, PFGE, sequencing
Plagiorchiida	ELISA, flotation, larvae migration method, sedimentation
Pneumocystales	culture
Pseudomonadales	API, culture, disk diffusion method, identification by Vitek automated system, IHC, MALDI-TOF MS, PCR
Reovirales	culture, HE stain, HIA, microscopy
Rhabditida	culture, flotation, histopathology, larvae migration method, microscopy, necropsy, parasitological coproscopy, parasitology, pathology, PCR, sedimentation, sequencing, urine sediment examination
Rickettsiales	blood smear, ELISA, IFA, IIFA, PCR, sequencing, WB
Serinales	biochemical examination, culture
Spirochaetales	culture, ELISA, IFA, IgG ELISA, IgM ELISA, IHA, IIFA, LIA, PCR, SNAP® 4D ×, WB
Trichinellida	digestion method, ELISA, IgG ELISA, IgM ELISA, magnetic stirrer method, PCR, RAPD, TEM, WB
Trypanosomatida	histology, histopathology, IFA, IHC, PCR, qPCR, sequencing, TEM
Vibrionales	culture, MALDI-TOF MS

*AGID* Agar gel immunodiffusion, *API* Analytical profile index, *CFT* Complement fixation test, *DFA assay* Direct fluorescent antibody assay, *DIA* DNA hybridization immunoassay, *ELISA* Enzyme-linked immunosorbent assay, *HE* Hematoxylin–eosin, *HIA* Hemagglutination inhibition assay, *IFA* Immunofluorescence assay, *Ig* Immunoglobulins, *IHA* Indirect hemagglutination assay, *IHC* Immunohistochemistry, *IIFA* Indirect immunofluorescence assay, *LAT* Latex agglutination test, *LIA* Line immunoassay, *MALDI-TOF MS* Matrix-assisted laser desorption/ionisation time-of-flight mass spectrometry, *MAT* Microscopic agglutination test, *MIFT* Microimmuno-fluorescence test, *MLSSR* Multilocus short sequence repeat, *MLST* Multilocus sequence typing, *M-VNTR* Mycobacterial interspersed repetitive unit-variable-number of tandem repeat, *NAT* Nucleic acid testing, *NI* Neuraminidase inhibition, *PAS* Periodic acid–Schiff reaction, *PCR* Polymerase chain reaction, *PFGE* Pulsed-field gel electrophoresis, *PRNT* Plaque reduction neutralisation assay, *RAPD* Random amplified polymorphic DNA, *RFLP* Restriction fragment length polymorphism, *RT-PCR* Reverse transcription polymerase chain reaction, *RT-qPCR* Reverse transcription quantitative polymerase chain reaction, *SNRA* Single nucleotide repeat analysis, *SRH* Single radial haemolysis test, *TEM* Transmission electron microscopy, *VNT* Virus neutralisation test, *WB* Western blot, *WGS* Whole genome sequencing

Serological methods were also common: enzyme-linked immunosorbent assay (ELISA) appeared in 69 (27%) studies, (indirect) immunofluorescence antibody (IFA/IFA) tests in 32 (12.5%), and the microscopic agglutination test (MAT, which is a *Leptospira*-specific serological test) in 21 (8.2%). Virus neutralization assays were used in 18 studies (7.0%), and plaque reduction neutralization tests (PRNT) in 12 (4.2%). Immunohistochemistry (IHC) and histopathology were each featured in nine studies (3.5%) (see Additional file 1 for a detailed breakdown of the frequency by technique).

Of the 256 studies reviewed, 202 used invasive sampling methods, 74 employed non-invasive sampling methods, and 27 involved environmental sampling (e.g., arthropods, surfaces, or faecal material collected from the ground), with some studies using more than one sample type. Regarding the condition of the equids sampled, 199 studies collected samples from live animals, 75 from deceased animals, and three relied on biobank archives. Since some studies used more than one sample type or source, these categories are not mutually exclusive.

#### Collaboration network

The co-authorship network comprised 1,233 nodes, each representing a unique author (see Additional file 3 for the co-authorship network visualization). Six authors were unconnected, indicating no collaborations within the network. The number of articles per author ranged from 1 to 8, with a median of 1 across the entire network; however, among the top 10 most prolific authors, the median number of publications was 6.5. The node degree distribution was right-skewed, denoting that while most authors collaborated with a few others (the most common number of collaborations (mode) was five), a small number of authors were highly collaborative (see Additional file 3 for the degree distribution). Notably, the authors with the highest number of collaborations were Veronesi Fabrizia (48 co-authorships), followed by Savini Giovanni (41), and Gehlen Heidrun (39). The mean number of co-authors per publication was 8.8 ± 5.9 (SD), with a median of 7.0.

**Fig. 6:**
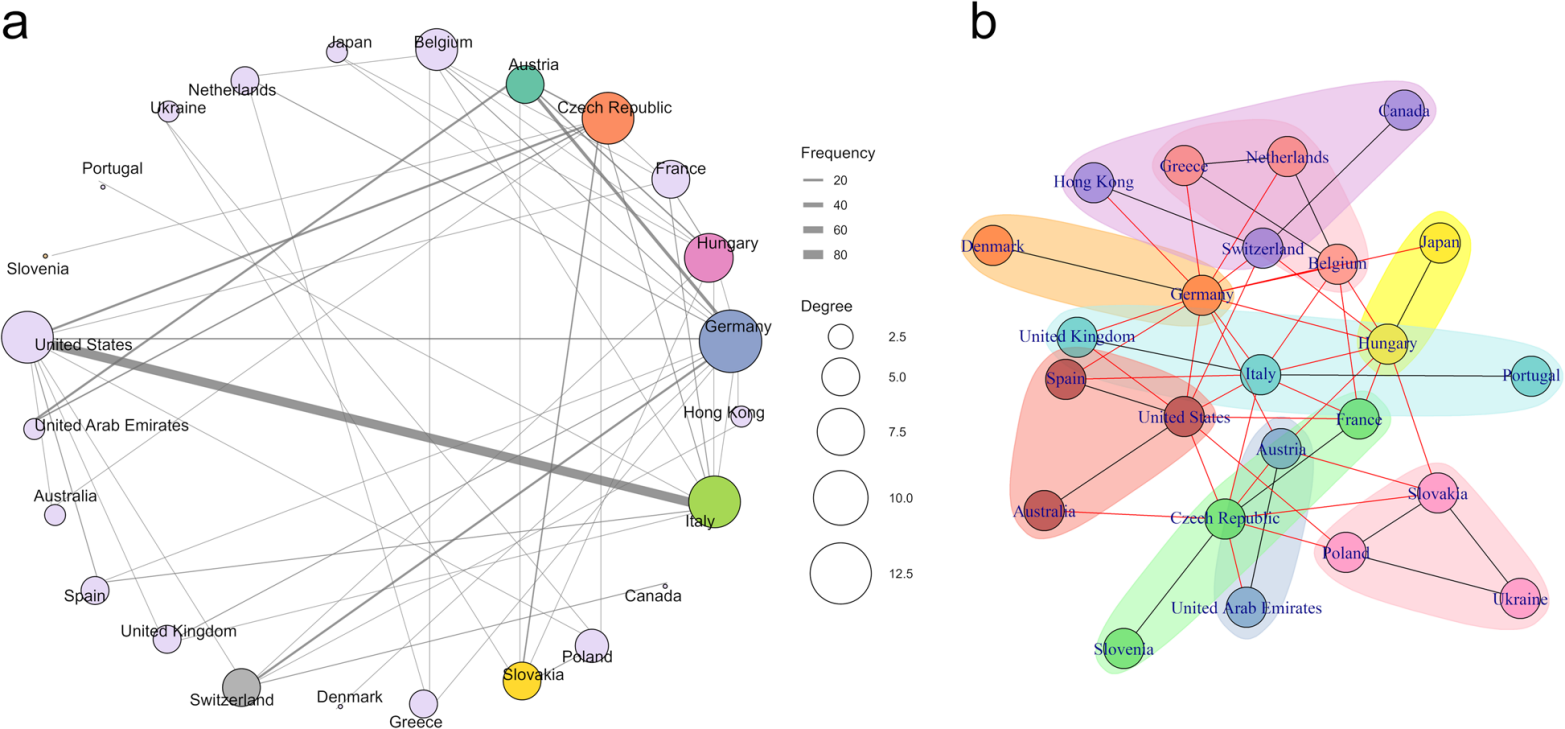
Country-level collaboration network on zoonotic equid diseases across Austria, the Czech Republic, Germany, Hungary, Italy, Slovenia, Slovakia, Liechtenstein, and Switzerland, 1964–2022. **a** Node size represents degree centrality (number of direct collaborators); edge width indicates collaboration frequency (co-publication count). **b** Node colour indicates communities detected using the Leiden algorithm, reflecting groups of countries that collaborated more frequently with each other than with other countries. Red edges show collaborations between clusters, while black edges represent collaborations within the same cluster

The collaboration network among countries included 24 nations, with an average of 1.3 ± 0.6 collaborating countries per article. Of the total publications, 57 (22.3%) were produced by multinational teams, averaging 2.3 ± 0.5 collaborating countries per paper. Germany led in multinational collaborations, partnering with 13 other countries within the network, followed by the Czech Republic, Italy, and the United States, each of which collaborated with nine countries. Notably, the strongest collaborative tie was between Italy and the United States (Fig. 6a). The Leiden algorithm identified nine communities of strongly connected countries. Examples of strong international collaborations included partnerships between Austria and the United Arab Emirates; the Czech Republic, France, and Slovenia; Italy, the United Kingdom, and Portugal; Germany and Denmark; Switzerland, Hong Kong, and Canada; Hungary and Japan; and Slovakia, Poland, and Ukraine (Fig. 6b).

### Detected zoonotic pathogens in equids in Central Europe

#### Diversity of detected zoonotic pathogens in equids

**Fig. 7:**
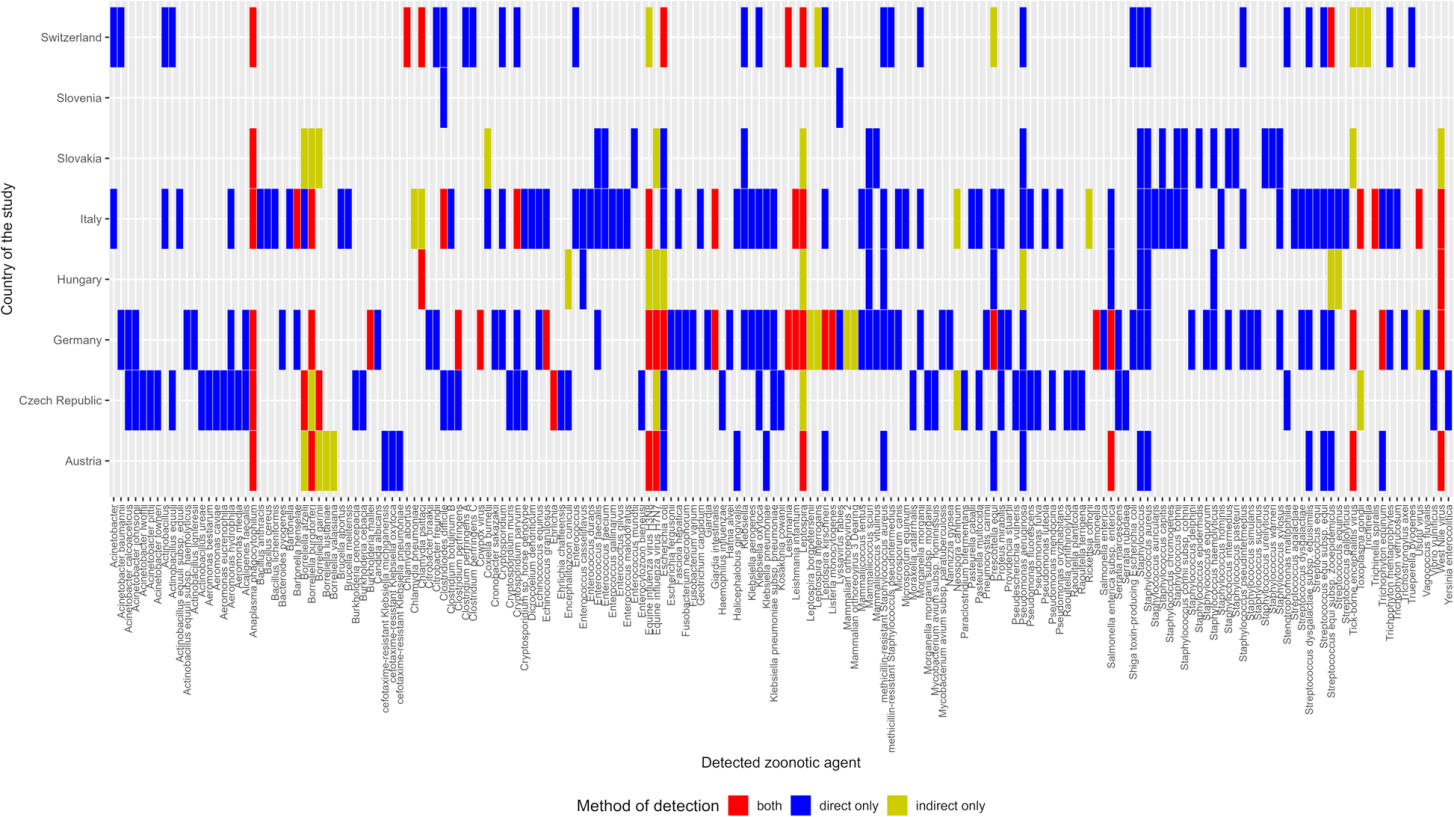
Detection methods used in 256 publications (1964–2022) on naturally occurring zoonotic pathogens in equids across Austria, the Czech Republic, Germany, Hungary, Italy, Slovenia, Slovakia, Liechtenstein, and Switzerland, categorised by direct, indirect, or both detection methods. No studies were reported from Liechtenstein

Out of the 191 zoonotic pathogens investigated in the region, 183 (95.8%) were successfully identified, of which 179 were evidenced in equids, whereas four (*Ehrlichia* spp.*, Ehrlichia chaffeensis*, *Trichophyton verrucosum*, and *Brucella melitensis*) were identified in humans solely (Table 1). Among the identified agents, 145 (79.2%) were bacteria, 29 (15.8%) were eukaryotes, and nine (4.9%) were viruses. Among these, 136 (74.3%) were evidenced solely by direct detection methods and were therefore considered as “circulating” in the region, while 37 (20.2%) were evidenced by both direct and indirect methods. Ten pathogens (5.5%), including *Borreliella lusitaniae*, *Borreliella valaisiana*, *Chlamydia pneumoniae*, *Leptospira interrogans*, *Leptospira borgpetersenii*, Mammalian orthoreovirus 2, Mammalian orthoreovirus 3 Dearing, *Neospora caninum*, *Rickettsia conorii*, and *Trichinella*, were detected solely by indirect methods (Fig. 7 and Additional file 1 for the breakdown of detection methods by superkingdom).

#### Geographic distribution

**Fig. 8:**
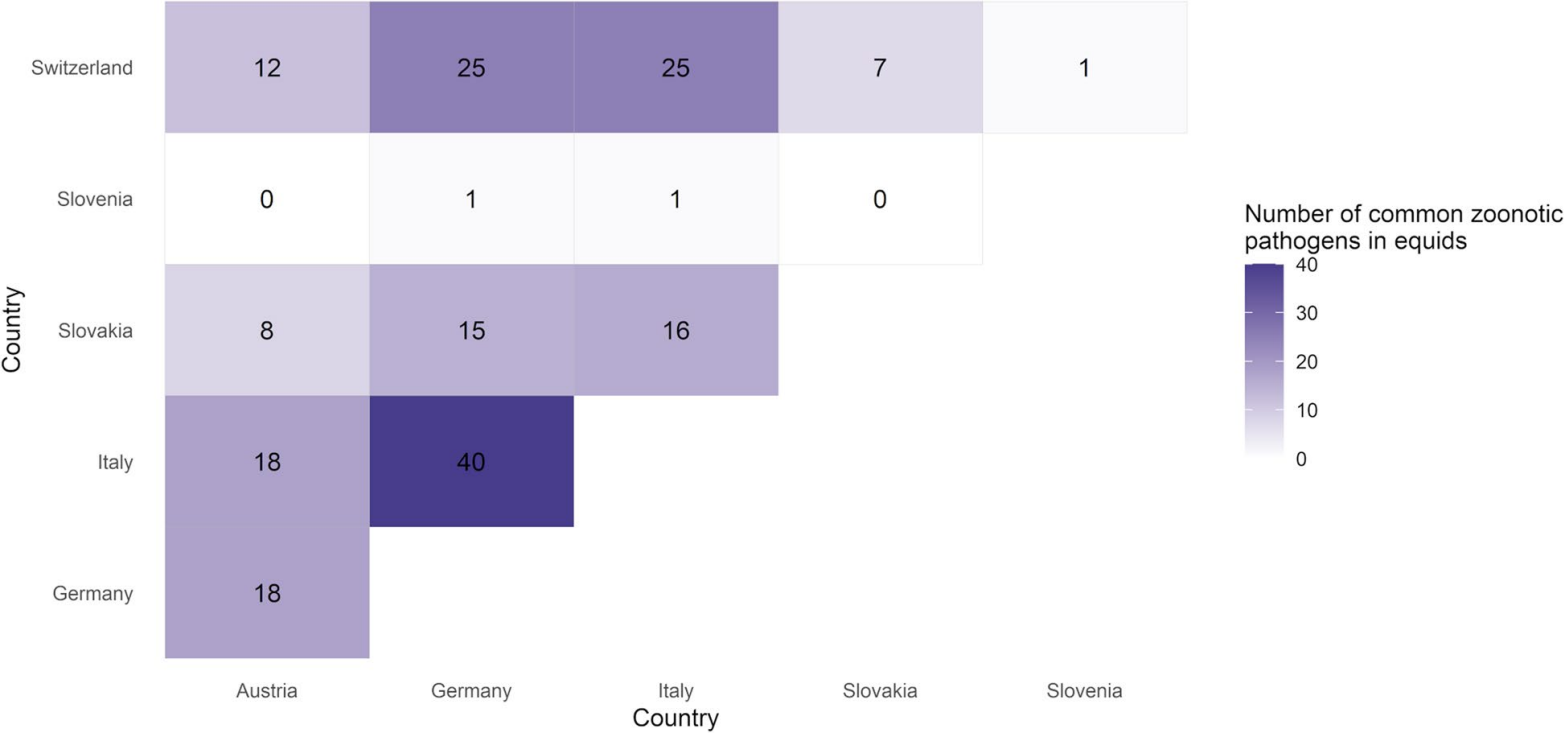
Distribution of shared detected zoonotic pathogens in equids across Austria, the Czech Republic, Germany, Hungary, Italy, Slovenia, Slovakia, Liechtenstein, and Switzerland, 1964–2022. Intensity of the colour represents the count of shared pathogens

Italy reported the largest number of equine zoonotic pathogens (*n* = 89), followed by Germany (*n* = 88), and the Czech Republic (*n* = 57) (Table 3). None of the detected pathogens were reported in all nine countries. However, pairwise comparisons of the detected zoonotic pathogens in equids revealed that Italy and Germany shared the highest number of pathogens, with 40 taxa in common. This was followed by Italy-Switzerland and Germany-Switzerland (25 shared taxa each) (Fig. 8).

**Table 3:** Distribution of identified zoonotic pathogens in equids by superkingdom across nine central European countries, Austria, the Czech Republic, Germany, Hungary, Italy, Slovenia, Slovakia, Liechtenstein, and Switzerland, 1964–2022. No studies were reported from Liechtenstein

Country of study	Bacteria	Eukaryota	Viruses
Austria	22	2	4
Czech Republic	49	7	1
Germany	63	16	9
Hungary	14	1	3
Italy	67	19	3
Slovakia	26	0	3
Slovenia	1	0	1
Switzerland	32	6	2

**Fig. 9:**
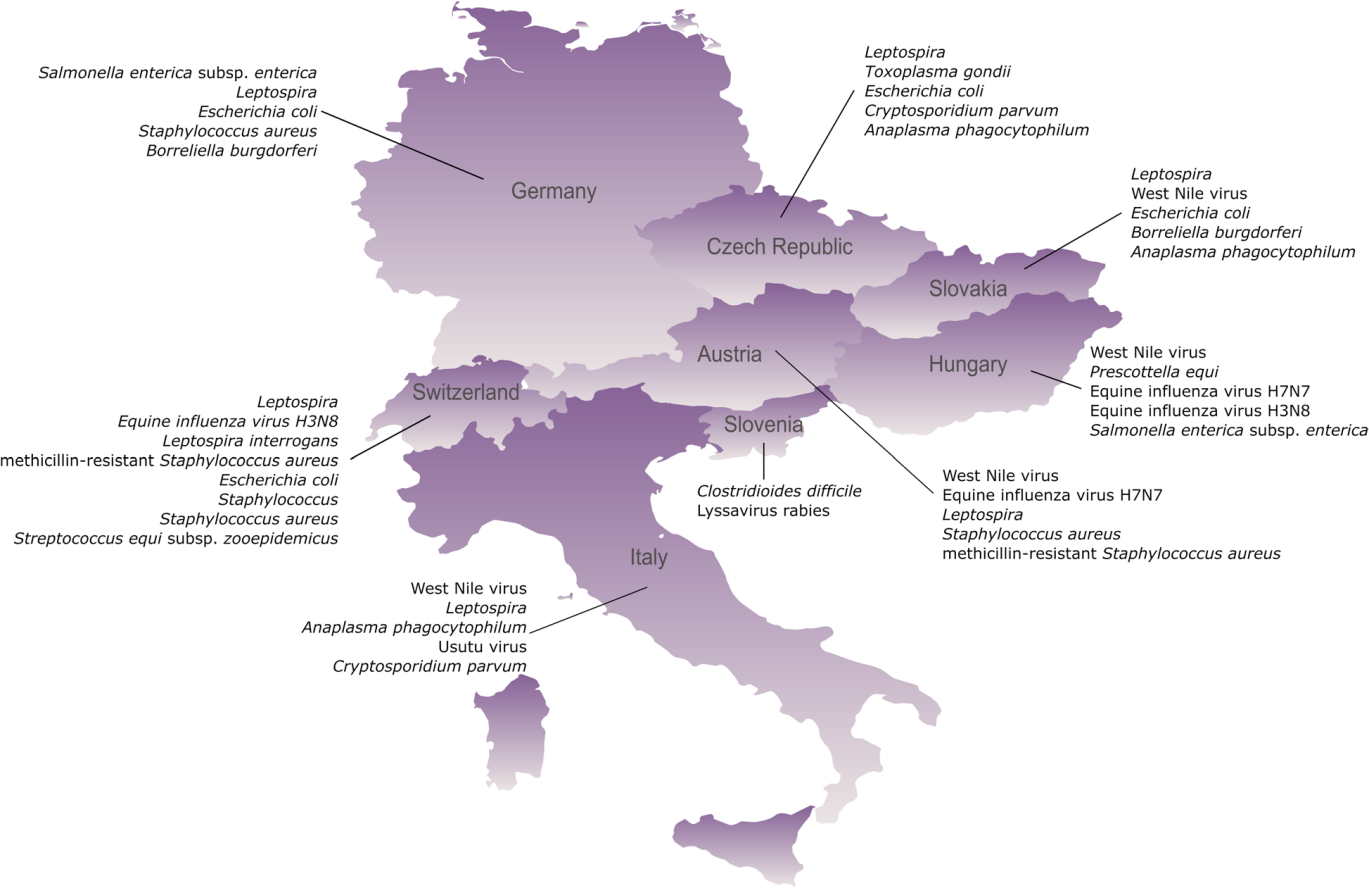
Top five most frequently reported zoonotic pathogens in equids, based on 256 publications (1964–2022) from Austria, the Czech Republic, Germany, Hungary, Italy, Slovenia, Slovakia, Liechtenstein, and Switzerland. No studies were reported from Liechtenstein

We identified the top five reported zoonotic pathogens in the literature for each of the nine countries (Fig. 9), highlighting notable variations in pathogen research focus across countries. Notably, *Leptospira* species appeared in the top five in six countries, whereas *E. coli* and WNV were among the top five in four countries, and *A. phagocytophilum* was included in the top five in three countries.

#### Diversity of hosts

**Fig. 10:**
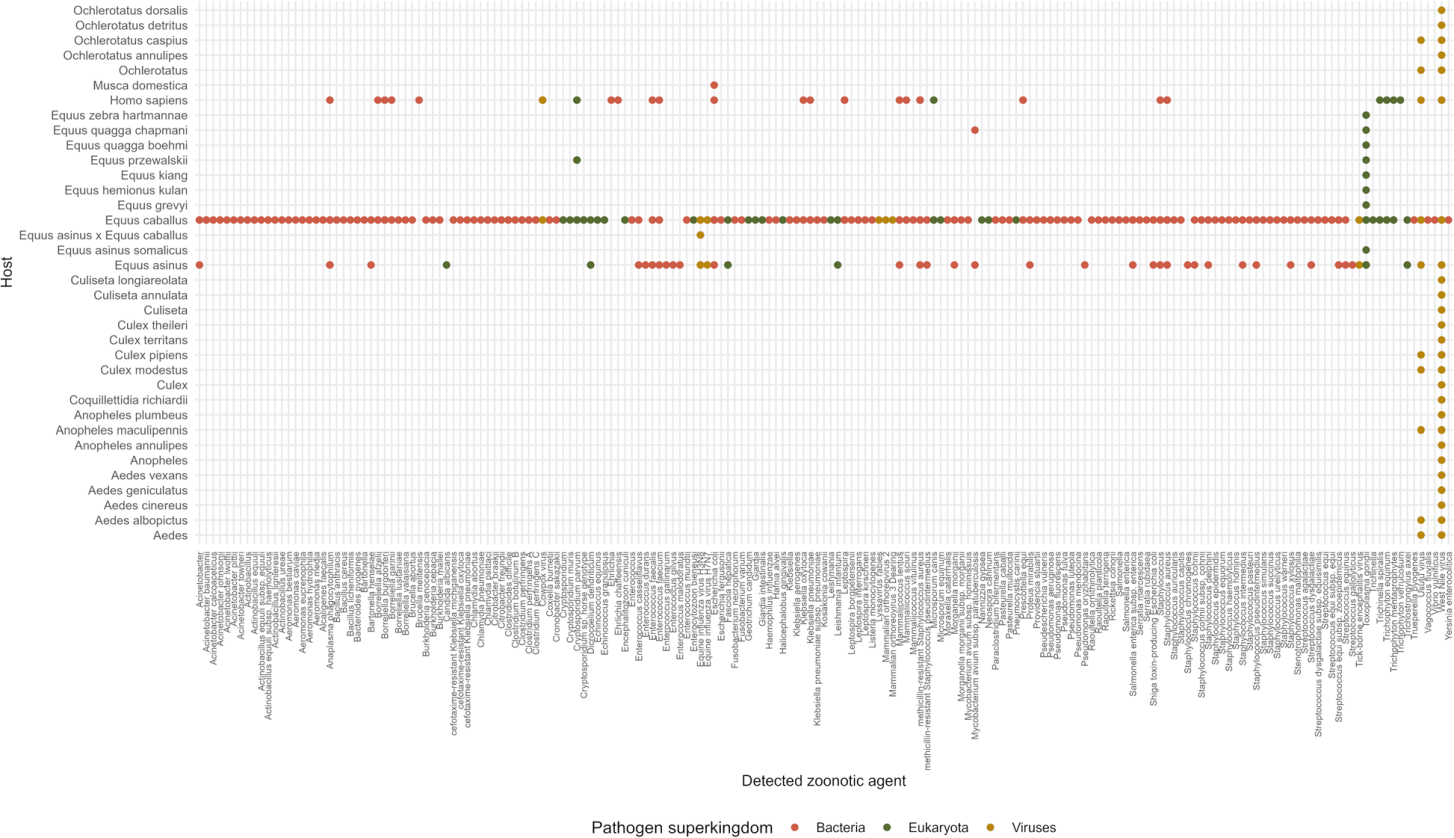
Distribution of detected equid-associated zoonotic pathogens across host species reported in 256 publications (1964–2022) from Austria, the Czech Republic, Germany, Hungary, Italy, Slovenia, Slovakia, Liechtenstein, and Switzerland, categorised by superkingdom

Across the reviewed studies, we identified 36 equine hosts, spanning nine taxonomic genera and four families: Equidae, with 11 species hosting 179 zoonotic agent taxa, Culicidae, with 23 species hosting two pathogen taxa, Hominidae, with one species (human), hosting 28 pathogen taxa, and Muscidae, with one species hosting one pathogen taxon. Within the Equidae family, horses (*Equus caballus*) were the most frequently identified hosts for equine zoonotic pathogens, with 170 taxa reported (92.9% of all zoonotic agent taxa), followed by asses (*Equus asinus*), with 43 taxa (23.5%) (Table 4, Fig. 10). A single zoonotic agent taxon can be found in multiple hosts.

**Table 4:** Number of zoonotic agent taxa identified using direct or indirect methods of detection across various hosts, as reported in 256 studies on equine zoonotic diseases conducted in Austria, the Czech Republic, Germany, Hungary, Italy, Slovenia, Slovakia, Liechtenstein, and Switzerland, 1964–2022. Hosts are listed in alphabetical order by their scientific names

NCBI-resolved scientific name of the host	NCBI-resolved name of the host	Number of zoonotic agent taxa reported	Percentage of the total number of unique zoonotic agent taxa
*Aedes*^1^	mosquito	2	1.1
*Aedes albopictus*	mosquito	2	1.1
*Aedes cinereus*	mosquito	1	0.5
*Aedes geniculatus*	mosquito	1	0.5
*Aedes vexans*	mosquito	1	0.5
*Anopheles*^1^	mosquito	1	0.5
*Anopheles annulipes*	mosquito	1	0.5
*Anopheles maculipennis*	mosquito	2	1.1
*Anopheles plumbeus*	mosquito	1	0.5
*Coquillettidia richiardii*	mosquito	1	0.5
*Culex*^1^	mosquito	1	0.5
*Culex modestus*	mosquito	2	1.1
*Culex pipiens*	mosquito	2	1.1
*Culex territans*	mosquito	1	0.5
*Culex theileri*	mosquito	1	0.5
*Culiseta*^1^	mosquito	1	0.5
*Culiseta annulata*	mosquito	1	0.5
*Culiseta longiareolata*	mosquito	1	0.5
*Equus asinus*	ass	43	23.5
*Equus asinus somalicus*	Somali Wild Ass	1	0.5
*Equus asinus x Equus caballus*	mule	1	0.5
*Equus caballus*	horse	170	92.9
*Equus grevyi*	Grevy's Zebra	1	0.5
*Equus hemionus kulan*	Kulan	1	0.5
*Equus kiang*	Tibetan Wild Ass	1	0.5
*Equus przewalskii*	Przewalski's horse	2	1.1
*Equus quagga boehmi*	Grant's Zebra	1	0.5
*Equus quagga chapmani*	Chapman's Zebra	2	1.1
*Equus zebra hartmannae*	Hartmann´s Mountain Zebra	1	0.5
*Homo sapiens*	human	28	15.3
*Musca domestica*	fly	1	0.5
*Ochlerotatus*^1^	mosquito	2	1.1
*Ochlerotatus annulipes*	mosquito	1	0.5
*Ochlerotatus caspius*	mosquito	2	1.1
*Ochlerotatus detritus*	mosquito	1	0.5
*Ochlerotatus dorsalis*	mosquito	1	0.5

^1^ Most specific scientific denomination extracted from the information source

#### Comparative analysis with the previously published list

While Sack et al. [22] reported identifying 56 zoonotic agent taxa, a closer examination of their table–particularly the disaggregation of broad categories such as “influenza virus A and B” into separate taxa (i.e., influenza A virus and influenza B virus)–revealed that the table lists 59 distinct taxa. A total of 74 genera were identified in the new dataset, versus 51 in Sack et al. [22], encompassing 40 common genera and 34 newly identified ones (Table 5). Conversely, 11 pathogens from Sack et al. [22], such as Hendra virus**,** Australian bat lyssavirus**,** Hepatitis E virus**,** and St. Louis encephalitis virus, remained absent in the current review.

**Table 5:** Comparison of zoonotic pathogen genera in equids between Sack et al. [22] and this review

Description	Count	List
Genera that are present in both studies (overlap)	40	*Acinetobacter, Actinobacillus, Alphainfluenzavirus, Anaplasma, Bacillus, Bartonella, Borreliella, Brucella, Burkholderia, Chlamydia, Clostridioides, Clostridium, Coxiella, Cryptosporidium, Echinococcus, Ehrlichia, Enterococcus, Enterocytozoon, Escherichia, Fasciola, Giardia, Halicephalobus, Klebsiella, Leishmania, Leptospira, Listeria, Lyssavirus, Microsporum, Mycobacterium, Orthoflavivirus, Orthopoxvirus, Prescottella, Rickettsia, Salmonella, Staphylococcus, Streptococcus, Toxoplasma, Trichinella, Trichophyton, Trichostrongylus*
Exclusive to Sack et al. (2020)^1^	11	*Alphavirus, Betainfluenzavirus, Blastocystis, Blastomyces, Campylobacter, Enterovirus, Henipavirus, Hepatitis E virus, Onchocerca, Parapoxvirus, Picobirnavirus*
Exclusive to this review^2^	34	*Aeromonas, Alcaligenes, Bacteroides, Candida, Citrobacter, Cronobacter, Dicrocoelium, Ectopseudomonas, Encephalitozoon, Fusobacterium, Geotrichum, Haemophilus, Hafnia, Kosakonia, Mammaliicoccus, Moraxella, Morganella, Nannizzia, Neospora, Orthoreovirus, Paraclostridium, Pasteurella, Pneumocystis, Proteus, Providencia, Pseudescherichia, Pseudomonas, Raoultella, Serratia, Stenotrophomonas, Trueperella, Vagococcus, Vibrio, Yersinia*

Genera identified in Sack et al. [22] but not in this review

^2^Genera identified in this review but not in Sack et al.[22]

## Discussion

This study provides a comprehensive overview of zoonotic pathogens in equids in Central Europe, spanning 58 years (1964–2022), and reveals that research on zoonotic equid pathogens is growing at a faster rate than the overall trend in scientific publications (8–9% each year [45]). A key strength of our approach is the rigorous validation of equid host, vector, and pathogen names, along with sharing the code for stepwise data analysis. This enhances transparency, allows for future updates, and facilitates comparisons with other geographic regions. Notably, 94.5% of the zoonotic agents catalogued in this review were detected through direct methods, providing definitive evidence of pathogen presence and strongly supporting the validity of our findings. The remaining 5.5% were identified through serological assays, where potential cross-reactivity could not be ruled out. However, this small proportion represents a minimal limitation that does not substantially impact the overall robustness of our conclusions regarding zoonotic threats to equids in Central Europe.

Our study identified 183 zoonotic taxa potentially infecting both equids and humans in Central Europe, representing a threefold increase over the 56 taxa reported by Sack et al. [22]. This discrepancy stems from selection criteria and underscores how methodological choices may shape zoonotic risk assessments. First, our taxonomic granularity extended beyond species level to include subspecies when available, whereas Sack et al. [22] aggregated pathogens at higher taxonomic levels. By preserving taxonomic granularity (genus, species, subspecies) in our dataset, we aimed to prevent oversight of emerging or cryptic zoonoses, enable reinterpretation of risks as evidence evolves, and reveal systemic gaps in pathogen reporting standards–a critical foundation for proactive One Health surveillance. Second, our host scope encompassed all members of the Equidae family (horses, donkeys, mules, zebras, and asses) whereas prior work focused solely on horses [22]. Third, we applied a broader technical definition of “equid zoonosis”, flagging any pathogen detected in equids with documented human infection, even opportunistic or hospital-acquired cases, while Sack et al. [22] restricted inclusion to pathogens with clear evidence of causing human disease, preferentially following equine exposure. Our inclusive definition follows strategies previously used in computational epidemiology [26, 27], facilitating hypothesis generation, prioritizing early risk identification, and enabling proactive surveillance. These differences collectively explain our expanded pathogen list and highlight the need for transparent, inclusive criteria in future zoonotic surveillance and One Health analyses.

By analysing research trends on equine zoonotic pathogens, we aimed to identify shifting research priorities and assess how scientific focus has evolved. Our findings revealed a marked intensification in regional research efforts beginning in the 1990s. The growing scientific focus on equid zoonoses was driven by increased research efforts in several countries within the region, particularly in Italy and Germany, with Austria, the Czech Republic, and Switzerland contributing to a lesser extent. This trend likely reflects global concerns about zoonotic and emerging infectious diseases [46–50], alongside increasing funding opportunities, highlighting that these pathogens directly affect animal health while posing serious risk to human health and the economy [51, 52]. Notably, the emergence of Hendra virus in Australia in 1994 [14], although not circulating in Europe, may have raised awareness that horses can play a role in the transmission of deadly zoonotic pathogens. Lastly, the rise in the number of publications may also be attributed to advances in detection methods and subsequent strengthened surveillance efforts [46]: for example, MALDI-TOF MS and WGS have greatly enhanced pathogen identification while next-generation sequencing (NGS) is an unbiased approach that enables the detection of all potential pathogens in a single assay without prior knowledge. However, NGS requires advanced bioinformatics, stringent filtering, and clinical expertise to differentiate disease-causing agents, resistance genes, and virulence factors from background noise [53–55].

The top 10 most studied zoonotic pathogens in equids accounted for almost two-thirds of the included regional literature. *Leptospira,* equine influenza virus H3N8, and *E. coli* have been studied over a long period, underscoring their consistent importance in the region. Equine influenza, which remains endemic in many parts of the world, is considered one of the most significant respiratory diseases affecting horses, with only a few countries–such as Iceland and New Zealand–remaining free of the disease [56]. While not typically life-threatening except in young foals, equine influenza can predispose infected horses to secondary bacterial infections, leading to severe and potentially fatal pneumonia. In addition, the virus is highly contagious, and outbreaks cause substantial economic losses for the equine industry [57]. Vaccination remains a key strategy in controlling its spread. Although transmission to humans is rare, when it occurs, it usually results in subclinical infections [58]. *Leptospira* is primarily transmitted through indirect contact with contaminated water or soil, typically through the urine of carrier animals like rats [59]. In horses, infection by pathogenic *Leptospira* may induce acute kidney injury and renal failure [60], abortion or stillbirth [61–64], and equine recurrent uveitis [65–67], with significant economic impacts on the horse industry [68].

Rising interest in tick-borne pathogens, such as *A. phagocytophilum* (the causative agent of human granulocytic anaplasmosis, HGA) [69–71] and *B. burgdorferi* (Lyme disease), reflects their increasing relevance in the temperate regions of the northern hemisphere [69, 72]. *A. phagocytophilum* causes equine granulocytic anaplasmosis (EGA) in horses, leading to thrombocytopenia, neutropenia, leukopenia, anaemia, and neurologic signs, with varying prevalence across regions [73, 74]. *S. equi* subsp. *zooepidemicus*, traditionally regarded as a commensal in animals, including horses and humans, has been increasingly associated since the 2000s with severe clinical outcomes in equids, humans, and dogs [75–78], including horse-to-human transmission [79]. Likewise, the emergence of MRSA as a significant human pathogen in Europe [80] and its growing recognition as a threat at human-horse interfaces [81–83] have likely contributed to heightened research interest. Equine professionals, such as veterinarians and stable workers, are at heightened risk of harbouring MRSA due to frequent contact with colonized horses or contaminated environments [84, 85].

In Europe, the emergence and further spread of the mosquito-borne orthoflaviviruses WNV and USUV have most probably driven recent publication increases. Both humans and horses are dead-end hosts for these viruses [86]. Although symptomatic infections in humans are rare, they can result in neurological disease and may be fatal [87]. Before the mid-1990s, human WNV infections in Europe were primarily sporadic, receiving limited research attention [87]. A particularly virulent outbreak in Romania in 1996 resulted in 393 human cases, with an estimated case fatality rate of approximately 4%, raising awareness of the disease [88]. Cases of WNV have now been reported in 35% of NUTS3 regions across eastern and central Europe. Projections for 2040–2060 suggest that WNV could affect between 44 and 56% of these areas, respectively [89]. In Austria alone, in 2024, there were 59 reported human WNV infections and 36 cases among equids, a significant increase compared to the single human and equine cases reported in 2023 [90]. Similarly, USUV was first detected outside of Africa in Austria, in 1996 [91], and by 2021, half of the EU/EEA countries had reported circulation of USUV in both humans and animals [92]. Evidence suggests that shifts in temperature and humidity may intensify vector-borne disease transmission [93–96]. During the 2000 WNV outbreak in southern France, the biting rate of the mosquito vector *Culex modestus* was strongly associated with rising temperature and increased rainfall [96, 97]. These trends highlight the necessity of integrating climate change considerations in zoonotic disease prioritization strategies. The One Health approach is crucial in addressing these challenges [92]. Furthermore, despite the growing importance of WNV, routine screening of blood products remains unestablished, which complicates blood donation management in affected areas [98].

Our analysis highlights a critical misalignment between ranking based on research effort and recognized public health risk for several key pathogens. Notably, *Brucella abortus*, *Brucella melitensis*, *Echinococcus multilocularis*, and *Echinococcus granulosus* (designated as high-priority threats in two frameworks) have received disproportionately low research attention in equid populations. This underinvestment may impede the development of effective surveillance and control measures for these pathogens. In contrast, 51 pathogens including *E. coli*, *S. aureus*, and *A. phagocytophilum*, dominated the literature despite their comparatively modest risk rankings. This discrepancy suggests that factors beyond evidence-based risk assessment, such as historical research trends, diagnostic convenience, or funding availability, may disproportionately shape research agendas. Addressing these gaps could enhance regional health security and preparedness.

Most studies employed invasive sampling methods, which can induce pain, stress, and other complications. We highlight this methodological consideration because zoo-housed equids (including endangered species like Przewalski’s horses or zebras) were occasionally included in the studies. Advancing and validating less- or non-invasive approaches [99–101] could improve routine monitoring of zoo populations, which face heightened ethical scrutiny and welfare risks from repeated invasive sampling, while maintaining diagnostic rigor.

Despite comprising 1,233 distinct authors from 24 countries, the co-authorship network was highly centralized: a small subset of authors maintained a disproportionately high number of collaborative ties, contrasting with the fewer connections of the majority, a pattern observed in other health research communities [102]. This disparity highlights a critical challenge for the field as scientific collaboration is a defining feature of contemporary academic research, with researchers increasingly working in teams to bring together diverse skills for shared goals while potentially reducing research costs and increasing productivity [103]. Collaboration is essential for accelerating innovation and driving societal impact through diverse expertise, culture, and resource integration [104, 105]. International collaborations emerged as a significant driver of research output, with 57 papers (22.3%) involving multinational teams. This aligns with broader trends in infectious disease research, where multi-country efforts are recognized as critical for tackling cross-border zoonotic threats, strengthening capacity, and swiftly addressing priority clinical research questions [106–108]. Germany and Italy stood out as central hubs in the network, likely reflecting their strategic roles in fostering cross-border partnerships. Commercial linkages, equine trade, and international equestrian events (e.g., competitions, breeding programs) may further drive communities of collaboration through shared economic and regulatory interests, or shared language and historical links [107, 109, 110].

A limitation of this study is our deliberate focus on equids in the search strategy, which excluded studies examining equid-associated pathogens solely in humans, vectors, or the environment. While this targeted approach allowed us to maintain a manageable scope and preserve the study's focus on equid-related pathogens, it inherently limits our understanding of the interactions and transmission dynamics at the human-equid-environment interface.

Yet very few studies reported the source or directionality of infection, and due to the heterogeneity and speculative nature of these reports, we did not systematically collect transmission‐route data. Additionally, only a limited number of studies utilized molecular or phylogenetic methods to trace transmission pathways, leaving critical knowledge gaps in understanding how pathogens spread. These gaps constrain our understanding of zoonotic spillover drivers. For instance, integrating human serosurveillance or genomic data could help confirm spillover events [111], refine zoonotic risk assessments, and clarify underreported pathways (e.g., foodborne or vector-borne transmission). Moreover, our broad inclusion criteria may overrepresent organisms without confirmed spillover. For example, *Acinetobacter towneri, Bacillus cereus, Cronobacter sakazakii* are environmental bacteria with limited evidence of equine-to-human transmission. Some of these pathogens thus should be viewed as indicators of potential zoonotic risk rather than confirmed zoonoses, warranting targeted epidemiological or experimental follow-up to confirm zoonotic pathways. Future reviews integrating human epidemiological data with equine and environmental surveillance data would strengthen the One Health relevance of such work.

Another limitation is our inclusion of pathogens at mixed taxonomic levels (genus, species, subspecies). For instance, *Actinobacillus* was counted separately from *Actinobacillus equuli,* which in turn was distinguished from *A. equuli subsp. equuli* and *A. equuli subsp. haemolyticus*. While this approach captures understudied taxa and highlights surveillance gaps, it may also overstate pathogen diversity, especially given inconsistent taxonomic reporting in the literature and despite our harmonization efforts.

Finally, our study did not incorporate a quality assessment of the included publications. Although this inclusive approach captures the breadth of equid zoonotic research, it also encompasses heterogeneous study designs, including cross-sectional surveys, case reports, surveillance data, longitudinal and case–control studies, all of which vary in methodological rigor [112]. This heterogeneity creates design-driven biases. One example is publication bias: case reports and observational studies are more likely to be published when they report novel or severe zoonotic events, skewing the literature toward rare or unusual findings while underreporting endemic or mild infections [113, 114]. Another example is selection bias: cross-sectional surveys may overrepresent accessible populations (e.g., clinical or farm settings), which may not reflect broader host populations or transmission dynamics. This can distort prevalence estimates and shape an incomplete or skewed picture of the range of known pathogens and their hosts [115, 116]. Furthermore, while we documented whether pathogens were identified via indirect (e.g., antibody detection) or direct methods (e.g., culture, PCR, WGS), we did not evaluate methodological rigor or evidence strength of these approaches. For example, detection methods vary in sensitivity and specificity. Nevertheless, given that our review remains descriptive (without a meta-analysis), the impact of this heterogeneity in study design and quality is likely limited [117]. Future work should incorporate quality assessment tools, like the Cochrane Risk-of-bias tool for randomised trials (RoB 2.0) [118], alongside systematic evaluation of methodological reliability (e.g., validation of assays, genomic resolution), to better assess the risk of bias and strengthen evidence-based prioritization of zoonotic threats in equids.

## Conclusions

Our systematic review demonstrates the expansion of equid zoonotic-disease research in Central Europe over the past six decades, most probably driven by heightened awareness of emerging pathogens and advances in detection technologies. WNV has received particular research attention, yet our analysis reveals that publication counts alone poorly reflect true zoonotic risk, as high-priority agents (e.g., *Brucella* spp., *Echinococcus* spp.) remain under-studied while lower-risk pathogens dominate the literature.

Geographic proximity and shared interests–whether scientific (e.g., aligned research priorities), strategic (e.g., biosecurity objectives), or economic (e.g., trade dependencies)–play a key role in shaping collaboration networks. These factors foster cross-border partnerships, which are critical for harmonizing surveillance systems, standardizing diagnostics, and coordinating response strategies, all essential for mitigating transboundary zoonotic threats. Moreover, the global growth of equestrian sports and trade elevates zoonotic exposure risks, reinforcing the need for One Health-driven prevention at human-equid interfaces. To close identified gaps, we recommend:

- Risk-aligned research prioritization: target high-impact but under-studied pathogens through dedicated funding and coordinated surveillance.
- Strengthened data-sharing frameworks: support open-access databases and partnerships to help countries share disease data more easily.
- Capacity building and network decentralization: support early-career and under-connected researchers via seed grants and exchange programs to diversify collaboration beyond established hubs.
- Integration of non-invasive diagnostics: validate and deploy less-invasive sampling methods to reduce animal stress while maintaining detection sensitivity.

Together, these measures will build a more innovative research and more effective framework for detecting and preventing zoonotic diseases in equids.

## Supplementary Information

Additional File 1.

Additional File 2.

Additional File 3.

## Data Availability

The datasets and R scripts supporting the conclusions of this article are available in the figshare repository DOI: 10.6084/m9.figshare.28399505, accessible at: https://figshare.com/s/ba2297316ec799781ca9.

## Abbreviations

AGID: Agar gel immunodiffusion
API: Analytical profile index
CABI: Centre for Agriculture and Bioscience International
CDC: Centers for Disease Control and Prevention
CFT: Complement fixation test
DFA assay: Direct fluorescent antibody assay
DIA: DNA hybridization immunoassay
DOI: Digital object identifier
EEA: European Economic Area
EGA: Equine granulocytic anaplasmosis
ELISA: Enzyme-linked immunosorbent assay
EU: European Union
HE: Hematoxylin–eosin
HGA: Human granulocytic anaplasmosis
HIA: Hemagglutination inhibition assay
IFA: Immunofluorescence assay
IHA: Indirect hemagglutination assay
IHC: Immunohistochemistry
IIFA: Indirect immunofluorescence assay
LAT: Latex agglutination test
LIA: Line immunoassay
MALDI-TOF MS: Matrix assisted laser desorption ionization-time of flight mass spectrometry
MAT: Microscopic agglutination test
MIFT: Microimmuno-fluorescence test
MLSSR: Multilocus short sequence repeat
MLST: Multilocus sequence typing
MRSA: Methicillin-resistant *Staphylococcus aureus*
M-VNTR: Mycobacterial interspersed repetitive unit-variable-number of tandem repeat
NAT: Nucleic acid testing
NCBI: National Center for Biotechnology Information
NGS: Next-generation sequencing
NI: Neuraminidase inhibition
NUTS3: Nomenclature of territorial units for statistics, level 3
PAS: Periodic acid-Schiff reaction
PCR: Polymerase-chain reaction
PFGE: Pulsed-field gel electrophoresis
PRISMA: Preferred Reporting Items for Systematic Review and Meta-Analysis
PRNT: Plaque reduction neutralisation assay
PROSPERO: International Prospective Register of Systematic Reviews
RAPD: Random amplified polymorphic DNA
RFLP: Restriction fragment length polymorphism
RT-PCR: Reverse transcription polymerase chain reaction
RT-qPCR: Reverse transcription quantitative polymerase chain reaction
SD: Standard deviation
OHZDP: One Health Zoonotic Disease Prioritization
SNRA: Single nucleotide repeat analysis
SRH: Single radial haemolysis test
TEM: Transmission electron microscopy
USUV: Usutu virus
VNT: Virus neutralisation test
WB: Western blot
WGS: Whole genome sequencing
WHO: World Health Organization
WNV: West Nile virus
WOAH: World Organisation for Animal Health

## References

[1] Librado P, Khan N, Fages A, Kusliy MA, Suchan T, Tonasso-Calvière L, et al. The origins and spread of domestic horses from the Western Eurasian steppes. Nature. 2021;598:634–40. 10.1038/s41586-021-04018-9. 34671162 10.1038/s41586-021-04018-9 PMC8550961

[2] Bonsi M, Anderson NE, Carder G. The socioeconomic impact of diseases of working equids in low and middle-income countries: A critical review. Animals. 2023;13:3865. 10.3390/ani13243865. 38136902 10.3390/ani13243865 PMC10741040

[3] Schoch CL, Ciufo S, Domrachev M, Hotton CL, Kannan S, Khovanskaya R, et al. NCBI Taxonomy: a comprehensive update on curation, resources and tools. 2020. Database. 10.1093/database/baaa062. 32761142 10.1093/database/baaa062 PMC7408187

[4] Food and Agriculture Organization of the United Nations. FAOSTAT: Crops and livestock products. https://www.fao.org/faostat/en/#data/QCL. Accessed 14 May 2024.

[5] World Horse Welfare, Eurogroup for Animals. Removing the Blinkers: The Health and Welfare of European Equidae in 2015. 2015. https://www.eurogroupforanimals.org/files/eurogroupforanimals/2021-12/EU-Equine-Report-Removing-the-Blinkers_0.pdf. Accessed 24 Apr 2025.

[6] Ministère de l'Agriculture et de la Souveraineté alimentaire (France). Infographie - La filière équine. 2024. https://agriculture.gouv.fr/infographie-la-filiere-equine. Accessed 2 Mar 2025.

[7] Ipsos. Pferdesport in Deutschland. 2020. https://www.pferd-aktuell.de/deutsche-reiterliche-vereinigung/zahlen--fakten. Accessed 2 Mar 2025.

[8] Federal Ministry of Agriculture, Forestry, Regions and Water Management Austria. Horse keeping in Austria. 2025. https://info.bml.gv.at/en/topics/agriculture/agriculture-in-austria/animal-production-in-austria/horse-keeping-in-austria.html. Accessed 19 Jun 2024.

[9] Joint WHO/FAO Expert Group on Zoonoses, World Health Organization & Food and Agriculture Organization of the United Nations. Joint WHO/FAO Expert Group on Zoonoses: bovine tuberculosis, Q fever, anthrax, psittacosis, hydatidosis, report on the first session. Geneva: World Health Organization; 1950. https://iris.who.int/handle/10665/40155.

[10] Karesh WB, Dobson A, Lloyd-Smith JO, Lubroth J, Dixon MA, Bennett M, et al. Ecology of zoonoses: natural and unnatural histories. Lancet. 2012;380:1936–45. 10.1016/S0140-6736(12)61678-X. 23200502 10.1016/S0140-6736(12)61678-X PMC7138068

[11] Hirst KM, Halsey SJ. Bacterial zoonoses impacts to conservation of wildlife populations: a global synthesis. Front Conserv Sci. 2023. 10.3389/fcosc.2023.1218153.

[12] Bernstein AS, Ando AW, Loch-Temzelides T, Vale MM, Li BV, Li H, et al. The costs and benefits of primary prevention of zoonotic pandemics. Sci Adv;8:eabl4183. 10.1126/sciadv.abl4183. 10.1126/sciadv.abl4183 PMC8816336 35119921

[13] O’Sullivan JD, Allworth AM, Paterson DL, Snow TM, Boots R, Gleeson LJ, et al. Fatal encephalitis due to novel paramyxovirus transmitted from horses. Lancet. 1997;349:93–5. 10.1016/s0140-6736(96)06162-4. 8996421 10.1016/s0140-6736(96)06162-4

[14] Murray K, Rogers R, Selvey L, Selleck P, Hyatt A, Gould A, et al. A novel morbillivirus pneumonia of horses and its transmission to humans. Emerg Infect Dis. 1995;1:31. 10.3201/eid0101.950107. 8903153 10.3201/eid0101.950107 PMC2626820

[15] European Food Safety Authority (EFSA) and European Center for Disease Prevention and Control. The European Union One Health. Zoonoses Report. EFSA J. 2022;2023(21): e8442. 10.2903/j.efsa.2023.8442 10.2903/j.efsa.2023.8442 PMC10714251 38089471

[16] World Health Organization (WHO), Food and Agriculture Organization of the United Nations (FAO), World Organisation for Animal Health (WOAH). Taking a Multisectoral, One Health Approach: A Tripartite Guide to Addressing Zoonotic Diseases in Countries: The Food and Agriculture Organization of the United Nations, The World Organisation for Animal Health, The World Health Organization; 2019.

[17] Ellwanger JH, Chies JAB. Zoonotic spillover: Understanding basic aspects for better prevention. Genet Mol Biol. 2021;44:e20200355. 10.1590/1678-4685-GMB-2020-0355. 34096963 10.1590/1678-4685-GMB-2020-0355 PMC8182890

[18] World Health Organization, editor. Global technical consultation report on proposed terminology for pathogens that transmit through the air. Geneva: World Health Organization; 2024. https://www.who.int/publications/m/item/global-technical-consultation-report-on-proposed-terminology-for-pathogens-that-transmit-through-the-air. Accessed 5 July 2025.

[19] Taylor K, Thomas S, Mendez D, Chicken C, Carrick J, Heller J, et al. “Prevention is the biggest success”: Barriers and enablers to personal biosecurity in the thoroughbred breeding industry. Prev Vet Med. 2020;183: 105135. 10.1016/j.prevetmed.2020.105135. 32961422 10.1016/j.prevetmed.2020.105135

[20] Chalmers RM, Salmon RL, Willshaw GA, Cheasty T, Looker N, Davies I, et al. Vero-cytotoxin-producing Escherichia coli O157 in a farmer handling horses. Lancet. 1997;349:1816. 10.1016/S0140-6736(05)61697-2 9269225 10.1016/s0140-6736(05)61697-2

[21] Venter M, Steyl J, Human S, Weyer J, Zaayman D, Blumberg L, et al. Transmission of West Nile virus during horse autopsy. Emerg Infect Dis. 2010;16:573–5. 10.3201/eid1603.091042. 20202454 10.3201/eid1603.091042 PMC3322023

[22] Sack A, Oladunni FS, Gonchigoo B, Chambers TM, Gray GC. Zoonotic diseases from horses: a systematic review. Vector Borne Zoonotic Dis. 2020;20:484–95. 10.1089/vbz.2019.2541. 32077811 10.1089/vbz.2019.2541 PMC7339018

[23] Page MJ, McKenzie JE, Bossuyt PM, Boutron I, Hoffmann TC, Mulrow CD, et al. The PRISMA 2020 statement: an updated guideline for reporting systematic reviews. Syst Rev. 2021;10:89. 10.1186/s13643-021-01626-4. 33781348 10.1186/s13643-021-01626-4 PMC8008539

[24] Ouzzani M, Hammady H, Fedorowicz Z, Elmagarmid A. Rayyan-a web and mobile app for systematic reviews. Syst Rev. 2016;5:210. 10.1186/s13643-016-0384-4. 27919275 10.1186/s13643-016-0384-4 PMC5139140

[25] Rivas-Ruiz F, Expósito-Ruiz M, Domínguez-Almendros S. Research designs in clinical epidemiology. Allergol Immunopathol. 2012;40:117–24. 10.1016/j.aller.2011.11.003. 10.1016/j.aller.2011.11.003 22284831

[26] Carlson CJ, Gibb RJ, Albery GF, Brierley L, Connor RP, Dallas TA, et al. The Global Virome in One Network (VIRION): an atlas of vertebrate-virus associations. mBio. 2022;13:e02985–21. 10.1128/mbio.02985-21. 10.1128/mbio.02985-21 PMC8941870 35229639

[27] Wardeh M, Sharkey KJ, Baylis M. Integration of shared-pathogen networks and machine learning reveals the key aspects of zoonoses and predicts mammalian reservoirs. Proc R Soc B Biol Sci. 2020;287:20192882. 10.1098/rspb.2019.2882. 10.1098/rspb.2019.2882 PMC7031665 32019444

[28] R Core Team. R: A language and environment for statistical computing. https://www.r-project.org/. Accessed 17 Nov 2024.

[29] Hadley W. Simple, consistent wrappers for common string operations [R package stringr]. 2023. https://cran.r-project.org/web/packages/stringr/index.html. Accessed 2 May 2025.

[30] Arshad A, Reif AH, Cavalleri J-MV, Desvars-Larrive A. Repository for the manuscript: "Zoonotic pathogens in equids in Central Europe: a systematic review". 1st ed. figshare; 2025. 10.1186/s12917-025-04915-5 PMC12235778 40629389

[31] Chamberlain SA, Szöcs E. Taxize: taxonomic search and retrieval in R. F1000. 2013;2:191. 10.12688/f1000research.2-191.v2. 10.12688/f1000research.2-191.v1 PMC3901538 24555091

[32] World Health Organization (WHO), R&D Blue Print. Pathogens Prioritization: A Scientific Framework for Epidemic and Pandemic Research Preparedness. 2024. https://www.who.int/publications/m/item/pathogens-prioritization-a-scientific-framework-for-epidemic-and-pandemic-research-preparedness. Accessed 2 May 2025

[33] World Organization for Animal Health. Chapter 1.3. Diseases, infections and infestations listed by WOAH. In: World Organization for Animal Health, editor. Terrestrial Code; 2024. https://www.woah.org/en/what-we-do/standards/codes-and-manuals/. Accessed 5 July 2025.

[34] Centers for Disease Control and Prevention (CDC). Completed OHZDP Workshops. 2024. https://www.cdc.gov/one-health/php/prioritization/completed-workshops.html#cdc_listing_res3-priority-zoonotic-diseases. Accessed 2 May 2025.

[35] Gábor Csárdi, T. Nepusz. The igraph software package for complex network research. 2006. https://igraph.org. Accessed 17 Nov 2024.

[36] Newman M. Networks: An Introduction: Oxford. UK: Oxford University Press, Inc; 2018.

[37] Traag VA, Waltman L, Van Eck NJ. From Louvain to Leiden: guaranteeing well-connected communities. Sci Rep. 2019;9:5233. 10.1038/s41598-019-41695-z. 30914743 10.1038/s41598-019-41695-z PMC6435756

[38] Radicchi F, Castellano C, Cecconi F, Loreto V, Parisi D. Defining and identifying communities in networks. PNAS. 2004;101:2658–63. 10.1073/pnas.0400054101. 14981240 10.1073/pnas.0400054101 PMC365677

[39] Wickham H. ggplot2: Elegant Graphics for Data Analysis. 2nd ed.: Springer; 2016.

[40] Pedersen TL. An implementation of grammar of graphics for graphs and networks [R package ggraph version 2.1.0]. 2024. https://cran.r-project.org/web/packages/ggraph/index.html. Accessed 2 May 2025.

[41] QGIS Development Team, Open Source Geospatial Foundation Project. QGIS Geographic Information System: Open Source Geospatial Foundation Project. 2024. http://www.qgis.org/. Accessed 14 Jan 2025.

[42] Dorneles EMS, Santana JA, Costa ACTRB, Junqueira DG, Heinemann MB, Lage AP. Equine brucellosis: current understanding and challenges. J Equine Vet Sci. 2023;127:104298. 10.1016/j.jevs.2023.104298. 37072072 10.1016/j.jevs.2023.104298

[43] Da Costa Vieira RF, Vieira, T S W., Nascimento, D. d. A. G., Martins TF, Krawczak FS, Labruna MB, et al. Serological survey of Ehrlichia species in dogs, horses and humans: zoonotic scenery in a rural settlement from southern Brazil. Rev Inst Med Trop S Paulo. 2013;55:335–40. 10.1590/S0036-46652013000500007. 10.1590/S0036-46652013000500007 PMC4105071 24037288

[44] Carmichael RC, Duell JR, Holbrook TC, Herrin BH, Leutenegger CM, O'Connor TP, et al. Antibodies reactive to Ehrlichia spp. are common in Oklahoma horses. Vector Borne Zoonotic Dis. 2014;14:552–6. 10.1089/vbz.2013.1570 10.1089/vbz.2013.1570 25072984

[45] Landhuis E. Scientific literature: Information overload. Nature. 2016;535:457–8. 10.1038/nj7612-457a. 27453968 10.1038/nj7612-457a

[46] Jones KE, Patel NG, Levy MA, Storeygard A, Balk D, Gittleman JL, et al. Global trends in emerging infectious diseases. Nature. 2008;451:990–3. 10.1038/nature06536 18288193 10.1038/nature06536 PMC5960580

[47] Ojeyinka OT, Omaghomi TT. Climate change and zoonotic diseases: a conceptual framework for predicting and managing health risks in the USA. GSC Biol Pharm Sci. 2024;26:27–36. 10.30574/gscbps.2024.26.3.0084.

[48] Desvars-Larrive A, Vogl AE, Puspitarani GA, Yang L, Joachim A, Käsbohrer A. A One Health framework for exploring zoonotic interactions demonstrated through a case study. Nat Commun. 2024;15:5650. 10.1038/s41467-024-49967-7. 39009576 10.1038/s41467-024-49967-7 PMC11250852

[49] Meadows AJ, Stephenson N, Madhav NK, Oppenheim B. Historical trends demonstrate a pattern of increasingly frequent and severe spillover events of high-consequence zoonotic viruses. BMJ Glob Health. 2023. 10.1136/bmjgh-2023-012026. 37918874 10.1136/bmjgh-2023-012026 PMC10626885

[50] Hossain L, Karimi F, Wigand RT, Crawford JW. Evolutionary longitudinal network dynamics of global zoonotic research. Scientometrics. 2015;103:337–53. 10.1007/s11192-015-1557-y. 32214547 10.1007/s11192-015-1557-y PMC7088546

[51] Stehr-Green JK, Schantz PM. The impact of zoonotic diseases transmitted by pets on human health and the economy. Vet Clin North Am Small Anim Pract. 1987;17:1–15. 10.1016/s0195-5616(87)50601-5. 3551300 10.1016/s0195-5616(87)50601-5

[52] Torgerson PR, Macpherson CNL. The socioeconomic burden of parasitic zoonoses: global trends. Vet Parasitol. 2011;182:79–95. 10.1016/j.vetpar.2011.07.017. 21862222 10.1016/j.vetpar.2011.07.017

[53] Gwinn M, MacCannell DR, Khabbaz RF. Integrating advanced molecular technologies into public health. J Clin Microbiol. 2017;55:703–14. 10.1128/JCM.01967-16. 28031438 10.1128/JCM.01967-16 PMC5328438

[54] Tan JK, Servellita V, Stryke D, Kelly E, Streithorst J, Sumimoto N, et al. Laboratory validation of a clinical metagenomic next-generation sequencing assay for respiratory virus detection and discovery. Nat Commun. 2024;15:9016. 10.1038/s41467-024-51470-y. 39532844 10.1038/s41467-024-51470-y PMC11558004

[55] Cito F, Di Francesco CE, Averaimo D, Chiaverini A, Alessiani A, Di Domenico M, et al. Streptococcus equi subsp. zooepidemicus: epidemiological and genomic findings of an emerging pathogen in central Italy. Animals 2025. 10.3390/ani15101351. 10.3390/ani15101351 PMC12108151 40427230

[56] Cullinane A, Elton D, Mumford J. Equine influenza – surveillance and control. Influenza Resp Viruses. 2010;4:339–44. 10.1111/j.1750-2659.2010.00176.x. 10.1111/j.1750-2659.2010.00176.x PMC4634605 20958927

[57] Smyth GB, Dagley K, Tainsh J. Insights into the economic consequences of the 2007 equine influenza outbreak in Australia. Austr Vet J. 2011;89:151–8. 10.1111/j.1751-0813.2011.00777.x. 10.1111/j.1751-0813.2011.00777.x 21711317

[58] Chambers TM. Equine Influenza. Cold Spring Harb Perspect Med. 2022;12:a038331. 10.1101/cshperspect.a038331. 32152243 10.1101/cshperspect.a038331 PMC8725621

[59] Levett PN. Leptospirosis. Clin Microbiol Rev. 2001;14:296–326. 10.1128/CMR.14.2.296-326.2001. 11292640 10.1128/CMR.14.2.296-326.2001 PMC88975

[60] Divers TJ. Acute kidney injury and renal failure in horses. Vet Clin N Am Equine Pract. 2022;38:13–24. 10.1016/j.cveq.2021.11.002. 10.1016/j.cveq.2021.11.002 35282961

[61] Cantón GJ, Navarro MA, Asin J, Chu P, Henderson EE, Mete A, et al. Equine abortion and stillbirth in California: a review of 1,774 cases received at a diagnostic laboratory, 1990–2022. J Vet Diagn Invest. 2023;35:153–62. 10.1177/10406387231152788. 36744759 10.1177/10406387231152788 PMC9999402

[62] Leon A, Pillon C, Tebourski I, Bruyas J-F, Lupo C. Overview of the causes of abortion in horses, their follow-up and management. Reprod Domest Anim. 2023;58(Suppl 2):93–101. 10.1111/rda.14406. 37312640 10.1111/rda.14406

[63] Di Azevedo MIN, Lilenbaum W. Equine genital leptospirosis: Evidence of an important silent chronic reproductive syndrome. Theriogenology. 2022;192:81–8. 10.1016/j.theriogenology.2022.08.029. 36063673 10.1016/j.theriogenology.2022.08.029

[64] Zečević I, Picardeau M, Vince S, Hađina S, Perharić M, Štritof Z, et al. Association between exposure to Leptospira spp. and abortion in mares in Croatia. Microorganisms. 2024;12:1039. 10.3390/microorganisms12061039. 10.3390/microorganisms12061039 PMC11205326 38930421

[65] Witkowski L, Cywinska A, Paschalis-Trela K, Crisman M, Kita J. Multiple etiologies of equine recurrent uveitis – A natural model for human autoimmune uveitis: A brief review. Comp Immunol Microbiol Infect Dis. 2016;44:14–20. 10.1016/j.cimid.2015.11.004. 26851589 10.1016/j.cimid.2015.11.004

[66] Arrieta-Bechara CE, Carrascal-Maldonado AY. Ocular leptospirosis: a review of current state of art of a neglected disease. Rom J Ophthalmol. 2022;66:282–8. 10.22336/rjo.2022.53. 36589326 10.22336/rjo.2022.53 PMC9773111

[67] Geiger T, Gerhards H, Bjelica B, Mackenthun E, Wollanke B. Analysis of 1840 equine intraocular fluid samples for the presence of anti-Leptospira antibodies and leptospiral DNA and the correlation to ophthalmologic findings in terms of Equine Recurrent Uveitis (ERU)-A retrospective study. Vet Sci. 2022;9:448. 10.3390/vetsci9080448. 36006363 10.3390/vetsci9080448 PMC9414351

[68] Spiess BM. Equine recurrent uveitis: The European viewpoint. Equine Vet J. 2010;42:50–6. 10.1111/j.2042-3306.2010.tb05635.x. 10.1111/j.2042-3306.2010.tb05635.x 20939167

[69] Parola P. Tick-borne rickettsial diseases: emerging risks in Europe. Comp Immunol Microbiol Infect Dis. 2004;27:297–304. 10.1016/j.cimid.2004.03.006. 15225980 10.1016/j.cimid.2004.03.006

[70] Alberti A, Addis MF, Sparagano O, Zobba R, Chessa B, Cubeddu T, et al. Anaplasma phagocytophilum, Sardinia. Italy Emerg Infect Dis. 2005;11:1322–4. 10.3201/eid1108.050085 16110587 10.3201/eid1108.050085 PMC3320504

[71] Chien RC, Mingqun L, Yan Q, Randolph N, Huang W, Wellman M, et al. Strains of Anaplasma phagocytophilum from horses in Ohio are related to isolates from humans in the northeastern USA. Microbiol Spectr. 2023;11: e0263223. 10.1128/spectrum.02632-23. 37882777 10.1128/spectrum.02632-23 PMC10715102

[72] Rizzoli A, Hauffe HC, Carpi G. Vourc’h GI, Neteler M, Rosà R. Lyme borreliosis in Europe Euro Surveill. 2011;16:19906. 10.2807/ese.16.27.19906-en. 21794218

[73] Bogdan AM, Mitrea IL, Ionita M. Equine granulocytic anaplasmosis: a systematic review and meta-analysis on clinico-pathological findings, diagnosis, and therapeutic management. Vet Sci. 2024;11:269. 10.3390/vetsci11060269. 38922016 10.3390/vetsci11060269 PMC11209296

[74] Aleman M, Vedavally U, Pusterla N, Wensley F, Berryhill E, Madigan JE. Common and atypical presentations of Anaplasma phagocytophilum infection in equids with emphasis on neurologic and muscle disease. J Vet Intern Med. 2024;38:440–8. 10.1111/jvim.16964. 38038253 10.1111/jvim.16964 PMC10800209

[75] Priestnall S, Erles K. Streptococcus zooepidemicus: an emerging canine pathogen. Vet J. 2011;188:142–8. 10.1016/j.tvjl.2010.04.028. 20570190 10.1016/j.tvjl.2010.04.028 PMC7110628

[76] Bosica S, Chiaverini A, Angelis ME, Petrini A, Averaimo D, Martino M, et al. Severe Streptococcus equi subspecies zooepidemicus outbreak from unpasteurized dairy product Consumption. Italy Emerg Infect Dis. 2023;29:1020–4. 10.3201/eid2905.221338. 37081588 10.3201/eid2905.221338 PMC10124651

[77] Cantelmi MC, Merola C, Averaimo D, Chiaverini A, Cito F, Cocco A, et al. Identification of the novel Streptococcus equi subsp. zooepidemicus sequence type 525 in donkeys of Abruzzo region, Italy. Pathogens. 2023;12:750. 10.3390/pathogens12060750. 10.3390/pathogens12060750 PMC10305129 37375440

[78] Lindahl SB, Aspán A, Båverud V, Paillot R, Pringle J, Rash NL, et al. Outbreak of upper respiratory disease in horses caused by Streptococcus equi subsp. zooepidemicus ST-24. Vet Microbiol. 2013;166:281–5. 10.1016/j.vetmic.2013.05.006. 10.1016/j.vetmic.2013.05.006 23773239

[79] Pelkonen S, Lindahl SB, Suomala P, Karhukorpi J, Vuorinen S, Koivula I, et al. Transmission of Streptococcus equi subspecies zooepidemicus infection from horses to humans. Emerg Infect Dis. 2013;19:1041–8. 10.3201/eid1907.121365. 23777752 10.3201/eid1907.121365 PMC3713971

[80] Stefani S, Varaldo PE. Epidemiology of methicillin-resistant staphylococci in Europe. Clin Microbiol Infect. 2003;9:1179–86. 10.1111/j.1469-0691.2003.00698.x. 14686982 10.1111/j.1469-0691.2003.00698.x

[81] Vincze S, Stamm I, Kopp PA, Hermes J, Adlhoch C, Semmler T, et al. Alarming proportions of methicillin-resistant Staphylococcus aureus (MRSA) in wound samples from companion animals, Germany 2010–2012. PLoS ONE. 2014;9:e85656. 10.1371/journal.pone.0085656. 24465637 10.1371/journal.pone.0085656 PMC3896405

[82] van Duijkeren E, Moleman M, van Sloet Oldruitenborgh-Oosterbaan MM, Multem J, Troelstra A, Fluit AC, et al. Methicillin-resistant Staphylococcus aureus in horses and horse personnel: an investigation of several outbreaks. Vet Microbiol. 2010;141:96–102. 10.1016/j.vetmic.2009.08.009. 19740613 10.1016/j.vetmic.2009.08.009

[83] Loeffler A, Lloyd DH. Companion animals: a reservoir for methicillin-resistant Staphylococcus aureus in the community? Epidemiol Infect. 2010;138:595–605. 10.1017/S0950268809991476. 20056014 10.1017/S0950268809991476

[84] Bullone M, Bellato A, Robino P, Nebbia P, Morello S, Marchis D, et al. Prevalence and risk factors associated with nasal carriage of methicillin-resistant staphylococci in horses and their caregivers. Vet Res (Veterinary Research). 2024;55:108. 10.1186/s13567-024-01364-0. 39252070 10.1186/s13567-024-01364-0 PMC11386249

[85] Jordan D, Simon J, Fury S, Moss S, Giffard P, Maiwald M, et al. Carriage of methicillin-resistant Staphylococcus aureus by veterinarians in Australia. Aust Vet J (Australian Veterinary Journal). 2011;89:152–9. 10.1111/j.1751-0813.2011.00710.x. 21495985 10.1111/j.1751-0813.2011.00710.x

[86] Kramer LD, Li J, Shi P-Y. West Nile virus. Lancet Neurol. 2007;6:171–81. 10.1016/S1474-4422(07)70030-3. 17239804 10.1016/S1474-4422(07)70030-3

[87] Reiter P. West Nile virus in Europe: understanding the present to gauge the future. Euro Surveill. 2010;15:19508. 10.2807/ese.15.10.19508-en. 20403311

[88] Tsai TF, Popovici F, Cernescu C, Campbell GL, Nedelcu NI. West Nile encephalitis epidemic in southeastern Romania. Lancet. 1998;352:767–71. 10.1016/s0140-6736(98)03538-7. 9737281 10.1016/s0140-6736(98)03538-7

[89] Farooq Z, Sjödin H, Semenza JC, Tozan Y, Sewe MO, Wallin J, et al. European projections of West Nile virus transmission under climate change scenarios. One Health. 2023;16:100509. 10.1016/j.onehlt.2023.100509. 37363233 10.1016/j.onehlt.2023.100509 PMC10288058

[90] Austrian Agency for Health and Food Safety. West Nile Virus. 09/01/2025. https://www.ages.at/en/human/disease/pathogens-from-a-to-z/west-nile-virus. Accessed 2 Mar 2025.

[91] Weissenböck H, Bakonyi T, Rossi G, Mani P, Nowotny N. Usutu virus, Italy, 1996. Emerg Infect Dis. 2013;19:274–7. 10.3201/eid1902.121191. 23347844 10.3201/eid1902.121191 PMC3559058

[92] Angeloni G, Bertola M, Lazzaro E, Morini M, Masi G, Sinigaglia A, et al. Epidemiology, surveillance and diagnosis of Usutu virus infection in the EU/EEA, 2012 to 2021. Euro Surveill. 2023;28:2200929. 10.2807/1560-7917.ES.2023.28.33.2200929. 37589592 10.2807/1560-7917.ES.2023.28.33.2200929 PMC10436690

[93] Caminade C, McIntyre KM, Jones AE. Impact of recent and future climate change on vector-borne diseases. Ann N Y Acad Sci. 2019;1436:157–73. 10.1111/nyas.13950. 30120891 10.1111/nyas.13950 PMC6378404

[94] Rupasinghe R, Chomel BB, Martínez-López B. Climate change and zoonoses: A review of the current status, knowledge gaps, and future trends. Acta Trop. 2022;226:106225. 10.1016/j.actatropica.2021.106225. 34758355 10.1016/j.actatropica.2021.106225

[95] Semenza JC, Menne B. Climate change and infectious diseases in Europe. Lancet Infect Dis. 2009;9:365–75. 10.1016/S1473-3099(09)70104-5. 19467476 10.1016/S1473-3099(09)70104-5

[96] Reiter P. Climate change and mosquito-borne disease: knowing the horse before hitching the cart. Revue scientifique et technique (International Office of Epizootics). 2008;27:383–98. 18819667

[97] Ludwig A, Bicout D, Chalvet-Monfray K, Sabatier P. Modelling the aggressiveness of the Culex modestus, possible vector of West Nile fever in Camargue, as a function of meteorological data. Environ Risque Santé (Environnement Risques Santé). 2005;4:109–13.

[98] Simonin Y. Circulation of West Nile virus and Usutu virus in Europe: Overview and challenges. Viruses. 2024;16:599. 10.3390/v16040599. 38675940 10.3390/v16040599 PMC11055060

[99] Lightbody KL, Davis PJ, Austin CJ. Validation of a novel saliva-based ELISA test for diagnosing tapeworm burden in horses. Vet Clin Pathol. 2016;45:335–46. 10.1111/vcp.12364. 27218436 10.1111/vcp.12364

[100] Share ER, Mastellar SL, Suagee-Bedore JK, Eastridge ML. Validation of a commercial ELISA kit for non-invasive measurement of equine cortisol concentration. J Equine Vet Sci. 2023;14:104309. 10.1016/j.jevs.2023.104309. 10.3390/ani14192831 PMC11475127 39409780

[101] Khan A, Olajide E, Friedrich M, Holt A, Goehring LS. Evaluation of non-invasive sampling techniques for the molecular surveillance of equid Herpesviruses in yearling horses. Viruses. 2024;16:1091. 10.3390/v16071091. 39066254 10.3390/v16071091 PMC11281437

[102] Yousefi-Nooraie R, Akbari-Kamrani M, Hanneman RA, Etemadi A. Association between co-authorship network and scientific productivity and impact indicators in academic medical research centers: a case study in Iran. Health Res Policy Syst. 2008;6:9. 10.1186/1478-4505-6-9. 18796149 10.1186/1478-4505-6-9 PMC2553770

[103] Fonseca BdP, Sampaio RB, Fonseca MV, Zicker F. Co-authorship network analysis in health research: method and potential use. Health Res Policy Syst (Health Research Policy and Systems). 2016;14:34. 10.1186/s12961-016-0104-5. 10.1186/s12961-016-0104-5 PMC4852432 27138279

[104] Research collaborations bring big rewards: the world needs more. Nature. 594:301–2. https://www.nature.com/articles/d41586-021-01581-z. 10.1038/d41586-021-01581-z 34135529

[105] Morel CM, Acharya T, Broun D, Dangi A, Elias C, Ganguly NK, et al. Health innovation networks to help developing countries address neglected diseases. Science. 2005;309:401–4. 10.1126/science.1115538. 16020723 10.1126/science.1115538

[106] Jit M, Ananthakrishnan A, McKee M, Wouters OJ, Beutels P, Teerawattananon Y. Multi-country collaboration in responding to global infectious disease threats: lessons for Europe from the COVID-19 pandemic. The Lancet Regional Health – Europe 2021. 10.1016/j.lanepe.2021.100221. 10.1016/j.lanepe.2021.100221 PMC8495250 34642675

[107] VanderWaal K, Deen J. Global trends in infectious diseases of swine. PNAS. 2018;115:11495–500. 10.1073/pnas.1806068115. 30348781 10.1073/pnas.1806068115 PMC6233110

[108] Fanning JP, Murthy S, Obonyo NG, Baillie JK, Webb S, Dalton HJ, et al. Global infectious disease research collaborations in crises: building capacity and inclusivity through cooperation. Glob Health. 2021;17:84. 10.1186/s12992-021-00731-2. 10.1186/s12992-021-00731-2 PMC8313114 34311748

[109] Hou L, Pan Y, Zhu JJ. Impact of scientific, economic, geopolitical, and cultural factors on international research collaboration. J Informetr. 2021;15:101194. 10.1016/j.joi.2021.101194.

[110] European Commission: Directorate-General for Research and Innovation, Manchester Institute of Innovation Research, Technopolis Group, Boekholt P, Cunningham P, et al. Drivers of international collaboration in research, Boekholt, P. (editor), Cunningham P. (editor) and Edler, J.(editor), Publications Office; 2009. https://op.europa.eu/en/publication-detail/-/publication/712e874d-4f61-4977-9512-3bb326c2ce63.

[111] Smith GJD, Vijaykrishna D, Bahl J, Lycett SJ, Worobey M, Pybus OG, et al. Origins and evolutionary genomics of the 2009 swine-origin H1N1 influenza A epidemic. Nature. 2009;459:1122–5. 10.1038/nature08182. 19516283 10.1038/nature08182

[112] Boutron I, Page MJ, Higgins JPT, Altman DG, Lundh A, Hróbjartsson A., on behalf of the Cochrane Bias Methods Group. Considering bias and conflicts of interest among the included studies: In Cochrane Handbook for Systematic Reviews of Interventions (eds J.P.T. Higgins, J. Thomas, J. Chandler, M. Cumpston, T. Li, M.J. Page and V.A. Welch): Cochrane; 2024.

[113] Thornton A, Lee P. Publication bias in meta-analysis: its causes and consequences. J Clin Epidemiol. 2000;53:207–16. 10.1016/S0895-4356(99)00161-4. 10729693 10.1016/s0895-4356(99)00161-4

[114] O'Brien SJ, Gillespie IA, Sivanesan MA, Elson R, Hughes C, Adak GK. Publication bias in foodborne outbreaks of infectious intestinal disease and its implications for evidence-based food policy. England and Wales 1992–2003. Epidemiol Infect. 2006;134:667–74. 10.1017/S0950268805005765 10.1017/S0950268805005765 PMC2870462 16420723

[115] Lines T, Burdick C, Dewez X, Aldridge E, Neal-Williams T, Walker K, et al. Nature and extent of selection bias resulting from convenience sampling in the emergency department. Emerg Med J. 2022;39:325. 10.1136/emermed-2021-211390. 34706898 10.1136/emermed-2021-211390

[116] Jager KJ, Tripepi G, Chesnaye NC, Dekker FW, Zoccali C, Stel VS. Where to look for the most frequent biases? Nephrology. 2020;25:435–41. 10.1111/nep.13706. 32133725 10.1111/nep.13706 PMC7318122

[117] McKenzie JE BSE. Synthesizing and presenting findings using other methods: In Cochrane Handbook for Systematic Reviews of Interventions (eds J.P.T. Higgins, J. Thomas, J. Chandler, M. Cumpston, T. Li, M.J. Page and V.A. Welch); 2019.

[118] Higgins JPT, Altman DG, Gøtzsche PC, Jüni P, Moher D, Oxman AD, et al. The Cochrane Collaboration’s tool for assessing risk of bias in randomised trials. BMJ. 2011;343:d5928. 10.1136/bmj.d5928. 22008217 10.1136/bmj.d5928 PMC3196245

