# Supplementary material for: Zoonotic pathogens in equids in Central Europe: a systematic review
Source: BMC Vet Res. 2025 Jul 8;21:451. doi: 10.1186/s12917-025-04915-5 (PMC12235778; doi:10.1186/s12917-025-04915-5)
Supplement: Supplementary file 1 — Additional File 1. [file 12917_2025_4915_MOESM1_ESM.pdf]

Additional file 1: Search queries, zoonotic references, and pathogen detection methods in equids research across Central Europe

Table 1 Search queries used in Scopus, CABI, and PubMed for the systematic review of zoonotic diseases in equids across Central Europe.

Database	Query
Scopus	Actinobacillus OR Acinetobacter OR "Anaplasma phagocytophilum" OR anaplasmosis OR lyssavirus OR "Bacillus anthracis" OR anthrax OR Bartonella OR bartonellosis OR Blastocystis OR Blastomyces OR "Borrelia burgdorferi" OR borreliosis OR Lyme OR "Botulinum toxin" OR botulism OR Brucella OR brucellosis OR "Burkholderia mallei" OR glanders OR Campylobacter* OR Chlamydia OR chlamydiosis OR "Clostridium difficile" OR clostridiosis OR clostridium OR "Coxiella burnetii" OR "Q fever" OR coxiellosis OR Cryptosporidium OR cryptosporidiosis OR Trichophyton OR trychophytosis OR dermatophyte OR Microsporum OR ringworm OR "tinea capitis" OR "tinea corporis" OR "Eastern equine encephalitis" OR "EEE" OR Echinococcus OR echinococcosis OR Ehrlichia OR ehrlichiosis OR Enterococc* OR Enterocytozoon OR Rhinovirus OR "Escherichia coli" OR "E. coli" OR "Fasciola hepatica" OR "liver fluke" OR fasciolasis OR Giardia OR giardiasis OR "Halicephalobus gingivalis" OR Hendra OR "Hepatitis E" OR Ilheus OR Influenza OR "Japanese encephalitis" OR Klebsiella OR Leishmania OR leishmaniasis OR Leptospira OR leptospirosis OR "recurrent uveitis" OR Listeria OR listeriosis OR "Methicillin-resistant staphylococcus" OR "Meticillin-resistant staphylococcus" OR "MRSA" OR Mycobacteri* OR Onchocerc* OR Parapox* OR Picobirnavirus OR Rabies OR "Rhodococcus equi" OR Rickettsia OR rickettsiosis OR Salmonell* OR SARS-CoV-2 OR COVID-19 OR Sindbis OR "St Louis encephalitis" OR "St. Louis encephalitis" OR "Saint Louis encephalitis" OR Staphylococcus OR Streptococc* OR strangles OR Toxoplasm* OR Trichinella OR trichinellosis OR trichinosis OR Trichostrongyl* OR Vaccina OR cowpox OR "Venezuelan equine encephalitis" OR VEE OR "West Nile" OR WNV AND Equidae OR equid OR equids OR equine OR Ass OR Asses OR Donkeys OR Equus OR Mules OR mule OR Zebras OR zebra OR horse OR horses AND Austria OR Germany OR Czech* OR Slovakia OR Hungary OR Slovenia OR Italy OR Switzerland OR Liechtenstein
CABI	((ab:Actinobacillus OR title:Actinobacillus OR ab:Acinetobacter OR title:Acinetobacter OR ab:"Anaplasma phagocytophilum" OR ab:anaplasmosis OR title:"Anaplasma phagocytophilum" OR title:anaplasmosis OR ab:lyssavirus OR

	title:lyssavirus OR ab:"Bacillus anthracis" OR ab:anthrax OR title:"Bacillus anthracis" OR title:anthrax OR ab:Bartonella OR ab:bartonellosis OR title:Bartonella OR title:bartonellosis OR ab:Blastocystis OR title:Blastocystis OR ab:Blastomyces OR title:Blastomyces OR ab:"Borrelia burgdorferi" OR ab:borreliosis OR ab:Lyme OR title:"Borrelia burgdorferi" OR title:borreliosis OR title:Lyme OR ab:"Botulinum toxin" OR ab:botulism OR title:"Botulinum toxin" OR title:botulism OR ab:Brucella OR ab:brucellosis OR title:Brucella OR title:brucellosis OR ab:"Burkholderia mallei" OR ab:glanders OR title:"Burkholderia mallei" OR title:glanders OR ab:Campylobacter* OR title:Campylobacter* OR ab:Chlamydia OR ab:chlamydiosis OR title:Chlamydia OR title:chlamydiosis OR ab:"Clostridium difficile" OR b:clostridiosis OR ab:clostridium OR title:"Clostridium difficile" OR title:clostridiosis OR title:clostridium OR ab:"Coxiella burnetii" OR ab:"Q fever" OR ab:coxiellosis OR title:"Coxiella burnetii" OR title:"Q fever" OR title:coxiellosis OR ab:Cryptosporidium OR ab:cryptosporidiosis OR title:Cryptosporidium OR title:cryptosporidiosis OR ab:Trichophyton OR ab:trychophytosis OR ab:dermatophyte OR title:Trichophyton OR title:trychophytosis OR title:dermatophyte OR ab:Microsporum OR ab:ringworm OR ab:"tinea capitis" OR ab:"tinea corporis" OR title:Microsporum OR title:ringworm OR title:"tinea capitis" OR title:"tinea corporis" OR ab:"Eastern equine encephalitis" OR ab:"EEE" OR title:"Eastern equine encephalitis" OR title:"EEE" OR ab:Echinococcus OR ab:echinococcosis OR title:Echinococcus OR title:echinococcosis OR ab:Ehrlichia OR ab:ehrlichiosis OR title:Ehrlichia OR title:ehrlichiosis OR ab:Enterococc* OR title:Enterococc* OR ab:Enterocytozoon OR title:Enterocytozoon OR ab:Rhinovirus OR title:Rhinovirus OR ab:"Escherichia coli" OR ab:"E. coli" OR title:"Escherichia coli" OR title:"E. coli" OR ab:"Fasciola hepatica" OR ab:"liver fluke" OR ab:fasciolasis OR title:"Fasciola hepatica" OR title:"liver fluke" OR title:fasciolasis OR ab:Giardia OR ab:giardiasis OR title:Giardia OR title:giardiasis OR ab:"Halicephalobus gingivalis" OR title:"Halicephalobus gingivalis" OR ab:Hendra OR title:Hendra OR ab:Hepatitis E OR title:Hepatitis E OR ab:Ilheus OR title:Ilheus OR ab:Influenza OR title:Influenza OR ab:"Japanese encephalitis" OR title:"Japanese encephalitis" OR ab:Klebsiella OR title:Klebsiella OR ab:Leishmania OR ab:leishmaniasis OR title:Leishmania OR title:leishmaniasis OR ab:Leptospira OR ab:leptospirosis OR ab:"recurrent uveitis" OR title:Leptospira OR title:leptospirosis OR title:"recurrent uveitis" OR ab:Listeria OR ab:listeriosis OR title:Listeria OR title:listeriosis OR ab:"Methicillin-resistant staphylococcus" OR ab:"Meticillin-resistant staphylococcus" OR ab:"MRSA" OR title:"Methicillin-

	resistant staphylococcus" OR title:"Meticillin-resistant staphylococcus" OR title:"MRSA" OR ab:Mycobacteri* OR title:Mycobacteri* OR ab:Onchocerc* OR title:Onchocerc* OR ab:Parapox* OR title:Parapox* OR ab:Picobirnavirus OR title:Picobirnavirus OR ab:Rabies OR title:Rabies OR ab:"Rhodococcus equi" OR title:"Rhodococcus equi" OR ab:Rickettsia OR ab:rickettsiosis OR title:Rickettsia OR title:rickettsiosis OR ab:Salmonell* OR title:Salmonell* OR ab:SARS-CoV-2 OR ab:COVID-19 OR title:SARS-CoV-2 OR title:COVID-19 OR ab:Sindbis OR title:Sindbis OR ab:"St Louis encephalitis" OR ab:"Saint Louis encephalitis" OR ab:"St. Louis encephalitis" OR title:"St Louis encephalitis" OR title:"Saint Louis encephalitis" OR title:"St. Louis encephalitis" OR ab:Staphylococcus OR title:Staphylococcus OR ab:Streptococc* OR ab:strangles OR title:Streptococc* OR title:strangles OR ab:Toxoplasm* OR title:Toxoplasm* OR ab:Trichinella OR ab:trichinellosis OR ab:trichinosis OR title:Trichinella OR title:trichinellosis OR title:trichinosis OR ab:Trichostrongyl* OR title:Trichostrongyl* OR ab:Vaccina OR ab:cowpox OR title:Vaccina OR title:cowpox OR ab:"Venezuelan equine encephalitis" OR ab:VEE OR title:"Venezuelan equine encephalitis" OR title:VEE OR ab:"West Nile" OR ab:WNV OR title:"West Nile" OR title:WNV)<br>AND (ab:Equidae OR title:Equidae OR ab:equid OR title:equid OR ab:equids OR title:equids OR ab:equine OR title:equine OR ab:Ass OR title:Ass OR ab:Asses OR title:Asses OR ab:Donkeys OR title:Donkeys OR ab:Equus OR title:Equus OR ab:Mules OR title:Mules OR ab:mule OR title:mule OR ab:Zebras OR title:Zebras OR ab:zebra OR title:zebra OR ab:horse OR title:horse OR ab:horses OR title:horses) AND (Austria OR Germany OR Czech* OR Slovakia OR Hungary OR Slovenia OR Italy OR Switzerland OR Liechtenstein) NOT (systematic review OR review OR Meta-Analysis OR Books OR Documents))
PubMed	(Actinobacillus[tiab] OR Acinetobacter[tiab] OR "Anaplasma phagocytophilum"[tiab] OR anaplasmosis[tiab] OR lyssavirus[tiab] OR "Bacillus anthracis"[tiab] OR anthrax[tiab] OR Bartonella[tiab] OR bartonellosis[tiab] OR Blastocystis[tiab] OR Blastomyces[tiab] OR "Borrelia burgdorferi"[tiab] OR borreliosis[tiab] OR Lyme[tiab] OR "Botulinum toxin"[tiab] OR botulism[tiab] OR Brucella[tiab] OR brucellosis[tiab] OR "Burkholderia mallei"[tiab] OR glanders[tiab] OR Campylobacter*[tiab] OR Chlamydia[tiab] OR chlamydiosis[tiab] OR "Clostridium difficile"[tiab] OR clostridiosis[tiab] OR clostridium[tiab] OR "Coxiella burnetii"[tiab] OR "Q fever"[tiab] OR coxiellosis[tiab] OR Cryptosporidium[tiab] OR cryptosporidiosis[tiab] OR Trichophyton[tiab] OR trychophytosis[tiab] OR dermatophyte[tiab] OR Microsporum[tiab] OR

	ringworm[tiab] OR “tinea capitis”[tiab] OR “tinea corporis”[tiab] OR “Eastern equine encephalitis”[tiab] OR “EEE”[tiab] OR Echinococcus[tiab] OR echinococcosis[tiab] OR Ehrlichia[tiab] OR ehrlichiosis[tiab] OR Enterococc*[tiab] OR Enterocytozoon[tiab] OR Rhinovirus[tiab] OR “Escherichia coli”[tiab] OR “E. coli”[tiab] OR “Fasciola hepatica”[tiab] OR “liver fluke”[tiab] OR fasciolasis[tiab] OR Giardia[tiab] OR giardiasis[tiab] OR “Halicephalobus gingivalis”[tiab] OR Hendra[tiab] OR Hepatitis E[tiab] OR Ilheus[tiab] OR Influenza[tiab] OR “Japanese encephalitis”[tiab] OR Klebsiella[tiab] OR Leishmania[tiab] OR leishmaniasis[tiab] OR Leptospira[tiab] OR leptospirosis[tiab] OR “recurrent uveitis”[tiab] OR Listeria[tiab] OR listeriosis[tiab] OR “Methicillin-resistant staphylococcus”[tiab] OR “Meticillin-resistant staphylococcus”[tiab] OR “MRSA”[tiab] OR Mycobacteri*[tiab] OR Onchocerc*[tiab] OR Parapox*[tiab] OR Picobirnavirus[tiab] OR Rabies[tiab] OR “Rhodococcus equi”[tiab] OR Rickettsia[tiab] OR rickettsiosis[tiab] OR Salmonell*[tiab] OR SARS-CoV-2[tiab] OR COVID-19[tiab] OR Sindbis[tiab] OR “St Louis encephalitis”[tiab] OR “Saint Louis encephalitis”[tiab] OR “St. Louis encephalitis”[tiab] OR Staphylococcus[tiab] OR Streptococc*[tiab] OR strangles[tiab] OR Toxoplasm*[tiab] OR Trichinella[tiab] OR trichinellosis[tiab] OR trichinosis[tiab] OR Trichostrongyl*[tiab] OR Vaccina[tiab] OR cowpox[tiab] OR “Venezuelan equine encephalitis”[tiab]OR VEE[tiab] OR “West Nile”[tiab] OR WNV[tiab]) AND Equidae[MeSH Terms] AND (Austria[TW] OR Germany[TW] OR Czech*[TW] OR Slovakia[TW] OR Hungary[TW] OR Slovenia[TW] OR Italy[TW] OR Switzerland[TW] OR Liechtenstein[TW]) NOT (systematic review[Publication Type] OR review[Publication Type] OR Meta-Analysis[Publication Type] OR Books[Publication Type] OR Documents[Publication Type])

Table 2 Supporting references on zoonotic potential. Only zoonotic agents of horses are included.

**Zoonotic agent**	**References**
Clostridium perfringens	[1]
Dicrocoelium dendriticum	[2]
Encephalitozoon cuniculi	[3]
Pasteurella multocida	[4, 5]
Serratia rubidaea	[6, 7]
Streptococcus agalactiae	[8]
Streptococcus equi subsp. *equi*	[9–11]
Tick-borne encephalitis virus	[12]
Usutu virus	[13, 14]
Yersinia enterocolitica	[15]
Aeromonas hydrophila	[16]
Aeromonas caviae	[16]
Aeromonas media	[16]
Aeromonas bestiarum	[17]
Aeromonas eucrenophila	[18]
Alcaligenes faecalis	[19]
Bacillus cereus	[20]
Bacillus licheniformis	[21]
Bacteroides pyogenes	[22, 23]
Borreliella afzelii	[24]
Borreliella garinii	[24]
Borreliella lusitaniae	[25]
Borreliella valaisiana	[26]
Burkholderia cepacia	[27]
Burkholderia cenocepacia	[28]
Candida albicans	[29]
Citrobacter braakii	[30]
Citrobacter freundii	[31, 32]
Pseudomonas luteola	[33]
Paraclostridium bifermentans	[34]
Cronobacter sakazakii	[35]
Escherichia fergusonii	[36]
Pseudescherichia vulneris	[37]

Pseudomonas oryzihabitans	[38, 39]
Fusobacterium necrophorum	[40, 41]
Fusobacterium varium	[42]
Geotrichum candidum	[43]
Haemophilus influenzae	[44, 45]
Hafnia alvei	[46]
Kosakonia cowanii	[47]
Morganella morganii	[48]
Morganella morganii subsp. *morganii*	[49]
Moraxella catarrhalis	[50]
Neospora caninum	[51–53]
Pasteurella caballi	[54]
Pneumocystis carinii	[55]
Proteus mirabilis	[56]
Providencia stuartii	[57, 58]
Pseudomonas aeruginosa	[59, 60]
Pseudomonas fluorescens	[61]
Pseudomonas fulva	[62, 63]
Pseudomonas mendocina	[64]
Raoultella planticola	[65–67]
Raoultella ornithinolytica	[68]
Raoultella terrigena	[69]
Mammalian orthoreovirus 2	[70]
Mammalian orthoreovirus 3 Dearing	[71]
Serratia marcescens	[72, 73]
Stenotrophomonas maltophilia	[74, 75]
Streptococcus dysgalactiae subsp. *equisimilis*	[76, 77]
Streptococcus equinus	[78]
Streptococcus gallolyticus	[79]
Trueperella pyogenes	[80]
Vagococcus fluvialis	[81]
Vibrio vulnificus	[82, 83]

Table 3 Frequency of methods used for pathogen detection in the reviewed studies. The count represents the number of studies using each method.

Standardized method name	Count
PCR	108
culture	80
IFA	31
IgM ELISA	27
IgG ELISA	26
MAT	21
sequencing	21
MLST	17
VNT	17
ELISA	15
RT-PCR	15
MALDI-TOF MS	14
PRNT	11
PFGE	10
API	9
IHC	9
histopathology	9
microscopy	9
qPCR	8
WGS	6
disk diffusion method	6
CFT	5
HIA	5
RT-qPCR	5
flotation	5
HE stain	4
Identification by Vitek automated system	4
TEM	4
WB	4
Ziehl-Nielsen stain	4
LAT	3
PCR-RFLP	3
blood smear	3
fluorescence microscopy	3
gel electrophoresis	3
histology	3
pathology	3
biochemical examination	2
faecal smear	2
conventional method	2
larvae migration method	2
necropsy	2
sedimentation	2
serology	2
spa typing	2
virus isolation	2
AGID	1
DFA assay	1
DIA	1
IHA	1
IIFA	1
IgA ELISA	1
IgG LAT	1
IgG PRNT	1
IgG VNT	1
LIA	1
M-VNTR	1
MIFT	1
MLSSR	1
NAT	1
NI	1
PAS	1
RAPD	1
RAPD-PCR	1
RFLP	1
RFLP-PCR	1
RNA-scope in situ hybridization	1
SN assay	1
SNAP® 4D×	1
SNRA	1
SRH	1
agglutinin lysin reaction	1
baermann technique	1
clinical signs	1
digestion method	1
electrosyneresis	1
fluoroscent brightener	1
Giemsa stain	1
Grocott stain	1
hair perforation test	1
kit ANAERO	1
rapid ID32E system	1
infection trial on mice	1
jameson method	1
lactophenol blue stain	1
lysotype	1
magnetic stirrer method	1
mallein test	1
merthiolate-iodine-formaldehyde concentration method	1
mouse bioassay	1
nicotinic acid test	1
optical immunoassay	1
paper electrophoresis	1
parasitological coproscopy	1
parasitology	1
rapid immunoassay	1
sven gard method	1
urease test	1
urine sediment examination	1
NA	12

AGID: Agar gel immunodiffusion; API: Analytical profile index; CFT: Complement fixation test; DFA assay: Direct fluorescent antibody assay; DIA: DNA hybridization immunoassay; ELISA: Enzyme-linked immunosorbent assay; HE: Hematoxylin-eosin; HIA: Hemagglutination inhibition assay;IFA: Immunofluorescence assay; Ig: Immunoglobulins; IHA: Indirect hemagglutination assay; IHC: Immunohistochemistry; IIFA: Indirect immunofluorescence assay; LAT: Latex agglutination test; LIA: Line immunoassay; MALDI-TOF MS: Matrix-assisted laser desorption/ionisation time-of-flight mass spectrometry; MAT: Microscopic agglutination test; MIFT: Microimmuno-fluorescence test; MLSSR: Multilocus short

sequence repeat; MLST: Multilocus sequence typing; M-VNTR: Mycobacterial interspersed repetitive unit-variable-number of tandem repeat; NA: Non available data; NAT: Nucleic acid testing; NI: Neuraminidase inhibition; PAS: Periodic acid–Schiff reaction; PCR: Polymerase chain reaction; PFGE: Pulsed-field gel electrophoresis; PRNT: Plaque reduction neutralisation assay; RAPD: Random amplified polymorphic DNA; ; RFLP: Restriction fragment length polymorphism; RT-PCR: Reverse transcription polymerase chain reaction; RT-qPCR: Reverse transcription quantitative polymerase chain reaction; SNRA: Single nucleotide repeat analysis; SRH: Single radial haemolysis test; TEM: Transmission electron microscopy; VNT: Virus neutralisation test; WB: Western blot; WGS: Whole genome sequencing.

Table 4 Distribution of pathogen detection method types (direct/indirect/both) by superkingdom. The table shows the number of pathogen taxa detected by direct only, indirect only, or both methods.

Superkingdom	Method of detection	Number of pathogens
Bacteria	Both direct and indirect	22
Bacteria	Direct only	117
Bacteria	Indirect only	6
Eukaryota	Both direct and indirect	9
Eukaryota	Direct only	18
Eukaryota	Indirect only	2
Viruses	Both direct and indirect	6
Viruses	Direct only	1
Viruses	Indirect only	2

References

1. Jones RL. Clostridial enterocolitis. Vet Clin N Am Equine Pract. 2000;16:471–85. doi:10.1016/S0749-0739(17)30090-1.
2. Hazlett M, Stalker M, Lake M, Peregrine A. Hepatic Dicrocoelium dendriticum infection in a miniature horse. The Canadian Veterinary Journal. 2018;59:863–5.
3. Magalhães TR, Pinto FF, Queiroga FL. A multidisciplinary review about Encephalitozoon cuniculi in a One Health perspective. Parasitol Res. 2022;121:2463–79. doi:10.1007/s00436-022-07562-z.
4. Peng Z, Liu J, Liang W, Wang F, Wang L, Wang X, et al. Development of an online tool for Pasteurella multocida genotyping and genotypes of Pasteurella multocida from different hosts. Front Vet Sci. 2021;8:771157. doi:10.3389/fvets.2021.771157.
5. Ujvári B, Weiczner R, Deim Z, Terhes G, Urbán E, Tóth AR, et al. Characterization of Pasteurella multocida strains isolated from human infections. Comp Immunol Microbiol Infect Dis. 2019;63:37–43. doi:10.1016/j.cimid.2018.12.008.
6. Karkey A, Joshi N, Chalise S, Joshi S, Shrestha S, Thi Nguyen TN, et al. Outbreaks of Serratia marcescens and Serratia rubidaea bacteremia in a central Kathmandu hospital following the 2015 earthquakes. Trans R Soc Trop Med Hyg. 2018;112:467–72. doi:10.1093/trstmh/try077.
7. Litterio ML, Arazi S, Hernández C, Lopardo H. Isolation of Serratia rubidaea from a mixed infection after a horse bite. Rev Argent Microbiol. 2012;44:272–4.
8. Tettelin H, Masignani V, Cieslewicz MJ, Eisen JA, Peterson S, Wessels MR, et al. Complete genome sequence and comparative genomic analysis of an emerging human pathogen, serotype V Streptococcus agalactiae. PNAS. 2002;99:12391–6. doi:10.1073/pnas.182380799.
9. Bohlman T, Waddell H, Schumaker B. A case of bacteremia and pneumonia caused by Streptococcus equi subspecies equi infection in a 70-year-old female following horse exposure in rural Wyoming. Ann Clin Microbiol Antimicrob. 2023;22:65. doi:10.1186/s12941-023-00602-1.
10. Torpiano P, Nestorova N, Vella C. Streptococcus equi subsp. equi meningitis, septicemia and subdural empyema in a child. IDCases. 2020;21:e00808. doi:10.1016/j.idcr.2020.e00808.
11. Brzezinski P, Chiriac A. A human case of strangles (equine distemper) with skin lesions. Indian J Dermatol Venereol Leprol. 2016;82:198–200. doi:10.4103/0378-6323.162320.
12. Kwasnik M, Rola J, Rozek W. Tick-Borne Encephalitis-Review of the Current Status. J Clin Med. 2023;12:6603. doi:10.3390/jcm12206603.
13. Grottola A, Marcacci M, Tagliazucchi S, Gennari W, Di Gennaro A, Orsini M, et al. Usutu virus infections in humans: a retrospective analysis in the municipality of Modena, Italy. Clin Microbiol Infect. 2017;23:33–7. doi:10.1016/j.cmi.2016.09.019.

14. Bakonyi T, Jungbauer C, Aberle SW, Kolodziejek J, Dimmel K, Stiasny K, et al. Usutu virus infections among blood donors, Austria, July and August 2017 - Raising awareness for diagnostic challenges. Euro Surveill. 2017;22:17–644. doi:10.2807/1560-7917.ES.2017.22.41.17-00644.
15. Fredriksson-Ahomaa M, Cernela N, Hächler H, Stephan R. Yersinia enterocolitica strains associated with human infections in Switzerland 2001-2010. Eur J Clin Microbiol Infect Dis. 2012;31:1543–50. doi:10.1007/s10096-011-1476-7.
16. Janda JM, Abbott SL. The genus Aeromonas: taxonomy, pathogenicity, and infection. Clin Microbiol Rev. 2010;23:35–73. doi:10.1128/CMR.00039-09.
17. Sinclair HA, Heney C, Sidjabat HE, George NM, Bergh H, Anuj SN, et al. Genotypic and phenotypic identification of Aeromonas species and CphA-mediated carbapenem resistance in Queensland, Australia. Diagn Microbiol Infect Dis. 2016;85:98–101. doi:10.1016/j.diagmicrobio.2016.02.005.
18. Aravena-Roman M, Chang BJ, Riley TV, Inglis, Timothy J. J. Phenotypic characteristics of human clinical and environmental Aeromonas in Western Australia. Pathology. 2011;43:350–6. doi:10.1097/PAT.0b013e3283463592.
19. Huang C. Extensively drug-resistant Alcaligenes faecalis infection. BMC Infect Dis. 2020;20:833. doi:10.1186/s12879-020-05557-8.
20. Motoi N, Ishida T, Nakano I, Akiyama N, Mitani K, Hirai H, et al. Necrotizing Bacillus cereus infection of the meninges without inflammatory reaction in a patient with acute myelogenous leukemia: a case report. Acta Neuropathol. 1997;93:301–5. doi:10.1007/s004010050618.
21. Haydushka IA, Markova N, Kirina V, Atanassova M. Recurrent sepsis due to bacillus licheniformis. J Glob Infect Dis. 2012;4:82–3. doi:10.4103/0974-777X.93768.
22. Lee HK, Walls G, Anderson G, Sullivan C, Wong CA. Prolonged Bacteroides pyogenes infection in a patient with multiple lung abscesses. Respirol Case Rep. 2024;12:e01314. doi:10.1002/rcr2.1314.
23. Lau JY, Korman TM, Yeung A, Streitberg R, Francis MJ, Graham M. Bacteroides pyogenes causing serious human wound infection from animal bites. Anaerobe. 2016;42:172–5. doi:10.1016/j.anaerobe.2016.10.008.
24. Strle F, Ruzić-Sabljić E, Cimperman J, Lotric-Furlan S, Maraspin V. Comparison of findings for patients with Borrelia garinii and Borrelia afzelii isolated from cerebrospinal fluid. Clin Infect Dis. 2006;43:704–10. doi:10.1086/506936.
25. Collares-Pereira M, Couceiro S, Franca I, Kurtenbach K, Schäfer SM, Vitorino L, et al. First isolation of Borrelia lusitaniae from a human patient. J Clin Microbiol. 2004;42:1316–8. doi:10.1128/JCM.42.3.1316-1318.2004.
26. Diza E, Papa A, Vezyri E, Tsounis S, Milonas I, Antoniadis A. Borrelia valaisiana in cerebrospinal fluid. Emerg Infect Dis. 2004;10:1692–3. doi:10.3201/eid1009.030439.
27. Lipuma JJ. Burkholderia cepacia complex as human pathogens. J Nematol. 2003;35:212–7.

28. Wallner A, King E, Ngonkeu, Eddy L. M., Moulin L, Béna G. Genomic analyses of Burkholderia cenocepacia reveal multiple species with differential host-adaptation to plants and humans. BMC Genomics. 2019;20:803. doi:10.1186/s12864-019-6186-z.
29. Kim J, Sudbery P. Candida albicans, a major human fungal pathogen. J Microbiol. 2011;49:171–7. doi:10.1007/s12275-011-1064-7.
30. Hirai J, Uechi K, Hagihara M, Sakanashi D, Kinjo T, Haranaga S, et al. Bacteremia due to Citrobacter braakii: A case report and literature review. J Infect Chemother. 2016;22:819–21. doi:10.1016/j.jiac.2016.07.003.
31. Bai L, Xia S, Lan R, Liu L, Ye C, Wang Y, et al. Isolation and characterization of cytotoxic, aggregative Citrobacter freundii. PLOS ONE. 2012;7:e33054. doi:10.1371/journal.pone.0033054.
32. Jabeen I, Islam S, Hassan A.K.M. Imrul, Tasnim Z, Shuvo SR. A brief insight into Citrobacter species - a growing threat to public health. Front Antibiot 2023. doi:10.3389/frabi.2023.1276982.
33. Ramana KV, Kareem MA, Sarada, C. H. V., Sebastian S, Lebaka R, Ratnamani MS, et al. Chryseomonas luteola bacteremia in a patient with left pyocele testis with Fournier's scrotal gangrene. Indian J Pathol Microbiol. 2010;53:568–9. doi:10.4103/0377-4929.68280.
34. Kolander SA, Cosgrove EM, Molavi A. Clostridial endocarditis. Report of a case caused by Clostridium bifermentans and review of the literature. Arch Intern Med. 1989;149:455–6. doi:10.1001/archinte.149.2.455.
35. Lepuschitz S, Ruppitsch W, Pekard-Amenitsch S, Forsythe SJ, Cormican M, Mach RL, et al. Multicenter Study of Cronobacter sakazakii Infections in Humans, Europe, 2017. Emerg Infect Dis. 2019;25:515–22. doi:10.3201/eid2503.181652.
36. Savini V, Catavitello C, Talia M, Manna A, Pompetti F, Favaro M, et al. Multidrug-resistant Escherichia fergusonii: a case of acute cystitis. J Clin Microbiol. 2008;46:1551–2. doi:10.1128/JCM.01210-07.
37. Jain S, Nagarjuna D, Gaind R, Chopra S, Debata PK, Dawar R, et al. Escherichia vulneris: an unusual cause of complicated diarrhoea and sepsis in an infant. A case report and review of literature. New Microbes New Infect. 2016;13:83–6. doi:10.1016/j.nmni.2016.07.002.
38. Bendig JW, Mayes PJ, Eyers DE, Holmes B, Chin TT. Flavimonas oryzihabitans (Pseudomonas oryzihabitans; CDC group Ve-2): an emerging pathogen in peritonitis related to continuous ambulatory peritoneal dialysis? J Clin Microbiol. 1989;27:217–8. doi:10.1128/jcm.27.1.217-218.1989.
39. Woo K-S, Choi J-L, Kim B-R, Kim J-E, Kim K-H, Kim J-M, et al. Outbreak of Pseudomonas oryzihabitans pseudobacteremia related to contaminated equipment in an emergency room of a tertiary hospital in Korea. J Infect Chemother. 2014;46:42–4. doi:10.3947/ic.2014.46.1.42.
40. Hall V, Duerden BI, Magee JT, Ryley HC, Brazier JS. A comparative study of Fusobacterium necrophorum strains from human and animal sources by phenotypic reactions, pyrolysis mass

spectrometry and SDS-PAGE. J Med Microbiol. 1997;46:865–71. doi:10.1099/00222615-46-10-865.

41. Hagelskjaer LH, Prag J, Malczynski J, Kristensen JH. Incidence and clinical epidemiology of necrobacillosis, including Lemierre's syndrome, in Denmark 1990-1995. Eur J Clin Microbiol Infect Dis. 1998;17:561–5. doi:10.1007/BF01708619.
42. Lee SJ, Baek YJ, Kim JN, Lee KH, Lee EH, Yeom JS, et al. Increasing Fusobacterium infections with Fusobacterium varium, an emerging pathogen. PLOS ONE. 2022;17:e0266610. doi:10.1371/journal.pone.0266610.
43. Keene S, Sarao MS, McDonald PJ, Veltman J. Cutaneous geotrichosis due to Geotrichum candidum in a burn patient. Access Microbiol. 2019;1:e000001. doi:10.1099/acmi.0.000001.
44. Murphy TF, Apicella MA. Nontypable Haemophilus influenzae: a review of clinical aspects, surface antigens, and the human immune response to infection. Rev Infect Dis. 1987;9:1–15. doi:10.1093/clinids/9.1.1.
45. Wallace RJ, Baker CJ, Quinones FJ, Hollis DG, Weaver RE, Wiss K. Nontypable Haemophilus influenzae (biotype 4) as a neonatal, maternal, and genital pathogen. Rev Infect Dis. 1983;5:123–36. doi:10.1093/clinids/5.1.123.
46. Albert MJ, Alam K, Islam M, Montanaro J, Rahaman AS, Haider K, et al. Hafnia alvei, a probable cause of diarrhea in humans. Infect Immun. 1991;59:1507–13. doi:10.1128/iai.59.4.1507-1513.1991.
47. Berinson B, Bellon E, Christner M, Both A, Aepfelbacher M, Rohde H. Identification of Kosakonia cowanii as a rare cause of acute cholecystitis: case report and review of the literature. BMC Infect Dis. 2020;20:366. doi:10.1186/s12879-020-05084-6.
48. Laupland KB, Paterson DL, Edwards F, Stewart AG, Harris, Patrick N. A. Morganella morganii, an Emerging Cause of Bloodstream Infections. Microbiol Spectr. 2022;10:e0056922. doi:10.1128/spectrum.00569-22.
49. Schultz E, Barraud O, Madec J-Y, Haenni M, Cloeckaert A, Ploy M-C, et al. Multidrug Resistance Salmonella Genomic Island 1 in a Morganella morganii subsp. morganii Human Clinical Isolate from France. mSphere 2017. doi:10.1128/mSphere.00118-17.
50. Murphy TF, Parameswaran GI. Moraxella catarrhalis, a human respiratory tract pathogen. Clin Infect Dis. 2009;49:124–31. doi:10.1086/599375.
51. Duarte PO, Oshiro LM, Zimmermann NP, Csordas BG, Dourado DM, Barros JC, et al. Serological and molecular detection of Neospora caninum and Toxoplasma gondii in human umbilical cord blood and placental tissue samples. Sci Rep. 2020;10:9043. doi:10.1038/s41598-020-65991-1.
52. Tranas J, Heinzen RA, Weiss LM, McAllister MM. Serological evidence of human infection with the protozoan Neospora caninum. Clin Diagn Lab Immunol. 1999;6:765–7. doi:10.1128/CDLI.6.5.765-767.1999.

53. Lobato J, Deise S, Mineo TP, Amaral J, Silvia Segundo GR, Costa-Cruz JM, et al. Detection of immunoglobulin G antibodies to Neospora caninum in humans: high seropositivity rates in patients who are infected by human immunodeficiency virus or have neurological disorders. Clin Vaccine Immunol. 2006;13:84–9. doi:10.1128/CVI.13.1.84-89.2006.
54. Escande F, Vallee E, Aubart F. Pasteurella caballi infection following a horse bite. Zentralbl Bakteriol. 1997;285:440–4. doi:10.1016/S0934-8840(97)80010-2.
55. Walzer PD. Immunological features of Pneumocystis carinii infection in humans. Clin Diagn Lab Immunol. 1999;6:149–55. doi:10.1128/CDLI.6.2.149-155.1999.
56. Burke JP, Ingall D, Klein JO, Gezon HM, Finland M. Proteus mirabilis infections in a hospital nursery traced to a human carrier. N Engl J Med. 1971;284:115–21. doi:10.1056/NEJM197101212840301.
57. Liu J, Wang R, Fang M. Clinical and drug resistance characteristics of Providencia stuartii infections in 76 patients. Int J Med Res. 2020;48:300060520962296. doi:10.1177/0300060520962296.
58. Guidone GHM, Cardozo JG, Silva LC, Sanches MS, Galhardi LCF, Kobayashi RKT, et al. Epidemiology and characterization of Providencia stuartii isolated from hospitalized patients in southern Brazil: a possible emerging pathogen. Access Microbiol. 2023;5:000652.v4. doi:10.1099/acmi.0.000652.v4.
59. Rossi E, La Rosa R, Bartell JA, Marvig RL, Haagensen, Janus A. J., Sommer LM, et al. Pseudomonas aeruginosa adaptation and evolution in patients with cystic fibrosis. Nat Rev Microbiol. 2021;19:331–42. doi:10.1038/s41579-020-00477-5.
60. Jurado-Martín I, Sainz-Mejías M, McClean S. Pseudomonas aeruginosa: an audacious pathogen with an adaptable Arsenal of Virulence Factors. Int J Mol Sci. 2021;22:3128. doi:10.3390/ijms22063128.
61. Hsueh PR, Teng LJ, Pan HJ, Chen YC, Sun CC, Ho SW, et al. Outbreak of Pseudomonas fluorescens bacteremia among oncology patients. J Clin Microbiol. 1998;36:2914–7. doi:10.1128/JCM.36.10.2914-2917.1998.
62. Almuzara MN, Vazquez M, Tanaka N, Turco M, Ramirez MS, Lopez EL, et al. First case of human infection due to Pseudomonas fulva, an environmental bacterium isolated from cerebrospinal fluid. J Clin Microbiol. 2010;48:660–4. doi:10.1128/JCM.01849-09.
63. Stark J. First case of non-traumatic community-acquired Pseudomonas fulva infection. Indian J Med Microbiol. 2022;40:317–8. doi:10.1016/j.ijmmb.2021.12.010.
64. Gani M, Rao S, Miller M, Scoular S. Pseudomonas mendocina bacteremia: a case study and review of literature. Am J Case Rep. 2019;20:453–8. doi:10.12659/AJCR.914360.
65. Bonnet E, Julia F, Giordano G, Lourtet-Hascoet J. Joint infection due to Raoultella planticola: first report. Infection. 2017;45:703–4. doi:10.1007/s15010-017-1006-3.

66. Howell C, Fakhoury J. A case of Raoultella planticola causing a urinary tract infection in a pediatric patient. Translational Pediatrics. 2017;6:102–3. doi:10.21037/tp.2017.04.02.
67. Skelton WP, Taylor Z, Hsu J. A rare case of Raoultella planticola urinary tract infection in an immunocompromised patient with multiple myeloma. IDCases. 2017;8:9–11. doi:10.1016/j.idcr.2017.02.002.
68. Seng P, Boushab BM, Romain F, Gouriet F, Bruder N, Martin C, et al. Emerging role of Raoultella ornithinolytica in human infections: a series of cases and review of the literature. IJID. 2016;45:65–71. doi:10.1016/j.ijid.2016.02.014.
69. Mal PB, Sarfaraz S, Herekar F, Ambreen R. Clinical manifestation and outcomes of multi-drug resistant (MDR) Raoultella terrigena infection - A case series at Indus Health Network, Karachi, Pakistan. IDCases. 2019;18:e00628. doi:10.1016/j.idcr.2019.e00628.
70. Ouattara LA, Barin F, Barthez MA, Bonnaud B, Roingeard P, Goudeau A, et al. Novel human reovirus isolated from children with acute necrotizing encephalopathy. Emerg Infect Dis. 2011;17:1436–44. doi:10.3201/eid1708.101528.
71. Rosa UA, Oliveira Ribeiro G de, Villanova F, Luchs A, Pádua Milagres FA de, Komninakis SV, et al. First identification of mammalian orthoreovirus type 3 by gut virome analysis in diarrheic child in Brazil. Sci Rep. 2019;9:18599. doi:10.1038/s41598-019-55216-5.
72. Maki DG, Hennekens CG, Phillips CW, Shaw WV, Bennett JV. Nosocomial urinary tract infection with Serratia marcescens: an epidemiologic study. J Infect Dis. 1973;128:579–87. doi:10.1093/infdis/128.5.579.
73. Okuda T, Endo N, Osada Y, Zen-Yoji H. Outbreak of nosocomial urinary tract infections caused by Serratia marcescens. J Clin Microbiol. 1984;20:691–5. doi:10.1128/jcm.20.4.691-695.1984.
74. Brooke JS. Stenotrophomonas maltophilia: an emerging global opportunistic pathogen. Clin Microbiol Rev. 2012;25:2–41. doi:10.1128/CMR.00019-11.
75. Jang TN, Wang FD, Wang LS, Liu CY, Liu IM. Xanthomonas maltophilia bacteremia: an analysis of 32 cases. J Formos Med Assoc. 1992;91:1170–6.
76. Rantala S. Streptococcus dysgalactiae subsp. equisimilis bacteremia: an emerging infection. Eur J Clin Microbiol Infect Dis. 2014;33:1303–10. doi:10.1007/s10096-014-2092-0.
77. Takahashi T, Ubukata K, Watanabe H. Invasive infection caused by Streptococcus dysgalactiae subsp. equisimilis: characteristics of strains and clinical features. J Infect Chemother. 2011;17:1–10. doi:10.1007/s10156-010-0084-2.
78. Klein RS, Catalano MT, Edberg SC, Casey JI. Streptococcus equinus septicemia: report of two cases and review of the literature. Am J Med Sci. 1980;279:99–103. doi:10.1097/00000441-198003000-00003.
79. Boleij A, Tjalsma H. The itinerary of Streptococcus gallolyticus infection in patients with colonic malignant disease. Lancet Infect Dis. 2013;13:719–24. doi:10.1016/S1473-3099(13)70107-5.

80. Stuby J, Lardelli P, Thurnheer CM, Blum MR, Frei AN. Trueperella pyogenes endocarditis in a Swiss farmer: a case report and review of the literature. BMC Infect Dis. 2023;23:821. doi:10.1186/s12879-023-08810-y.
81. Jadhav KP, Pai PG. A rare infective endocarditis caused by Vagococcus fluvialis. J Cardiol Cases. 2019;20:129–31. doi:10.1016/j.jccase.2019.07.001.
82. Phillips KE, Satchell, Karla J. F. Vibrio vulnificus: From Oyster Colonist to Human Pathogen. PLoS Pathog. 2017;13:e1006053. doi:10.1371/journal.ppat.1006053.
83. Baker-Austin C, Oliver JD. Vibrio vulnificus: new insights into a deadly opportunistic pathogen. Environ Microbiol. 2018;20:423–30. doi:10.1111/1462-2920.13955.
