# Supplementary material for: Zoonotic pathogens in equids in Central Europe: a systematic review
Source: BMC Vet Res. 2025 Jul 8;21:451. doi: 10.1186/s12917-025-04915-5 (PMC12235778; doi:10.1186/s12917-025-04915-5)

Additional file 3: Co-authorship network and degree distribution analysis of scientists researching zoonotic diseases in equids across Central Europe (1964–2022)

Figure 1 Co-authorship network depicting collaboration among 1227 scientists publishing on zoonotic diseases of equids 1964-2022 and belonging to nine countries in Central Europe, Austria, the Czech Republic, Germany, Hungary, Italy, Slovenia, Slovakia, Liechtenstein, and Switzerland. Nodes represent authors; edges represent co-authorship. Node size represent the degree centrality, i.e., the number of links a node has, which represent the number of co-authorship or co-production between authors of a given publication. Isolated nodes are not shown (six authors).

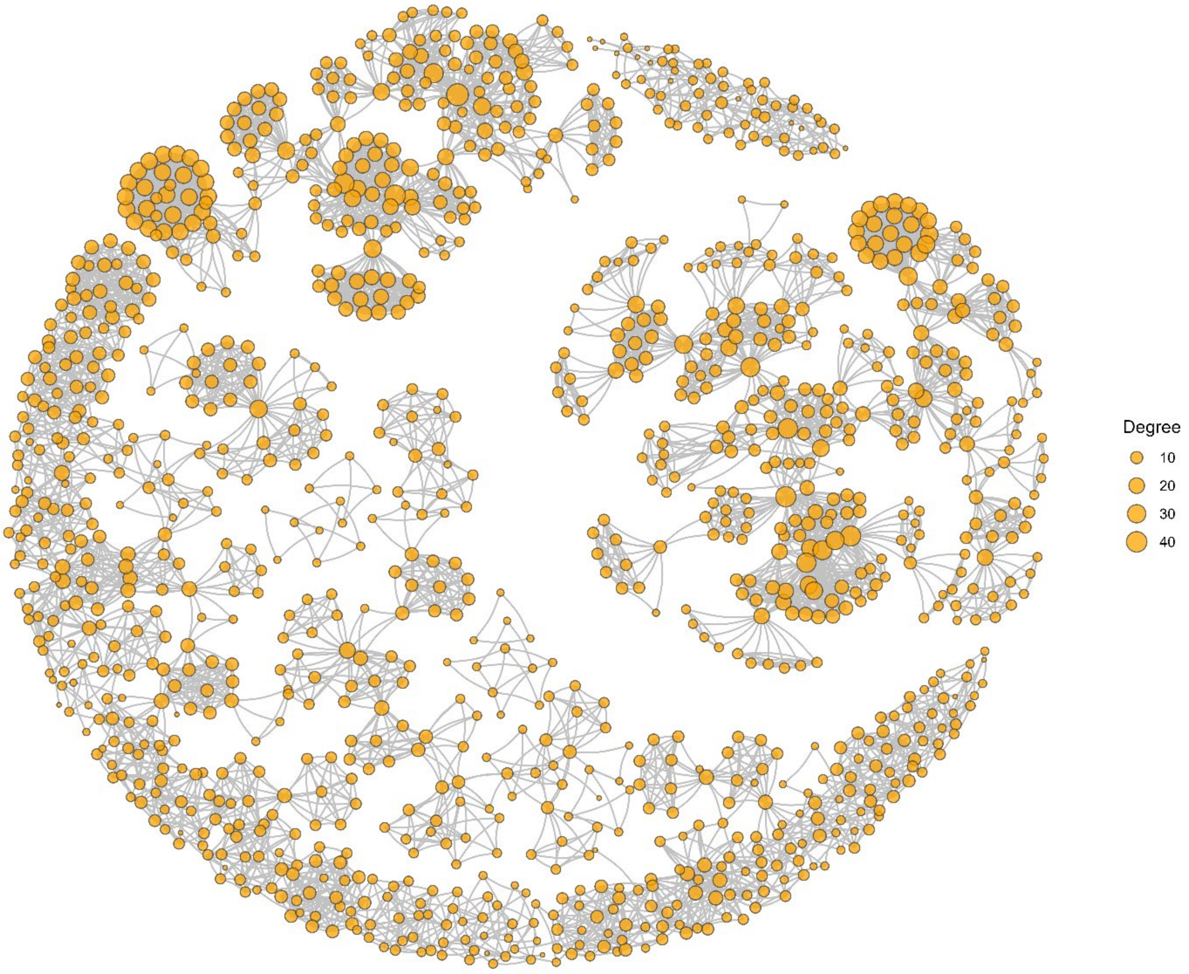

Figure 2 Degree distribution in the co-authorship network among 1227 scientists from nine Central European countries (Austria, the Czech Republic, Germany, Hungary, Italy, Slovenia, Slovakia, Liechtenstein, and Switzerland) publishing on zoonotic diseases of equids. The degree distribution represents the number of co-authorship connections (i.e., the number of collaborators) each scientist has within the network, highlighting the level of collaboration and the centrality of researchers in the field.

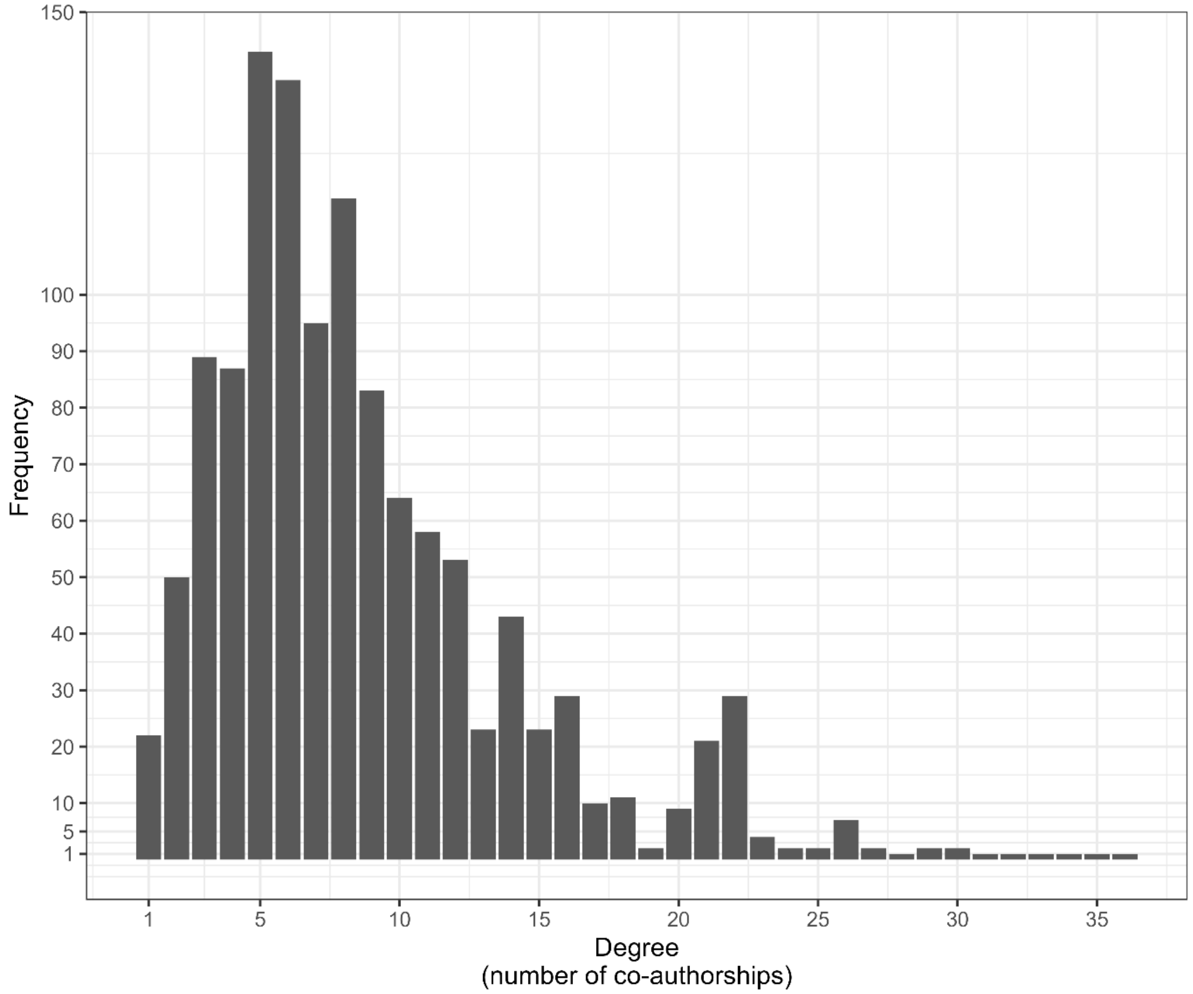

Supplement: Supplementary file 3 — Additional File 3. [file 12917_2025_4915_MOESM3_ESM.pdf]
